# An Active Type I-E CRISPR-Cas System Identified in *Streptomyces avermitilis*

**DOI:** 10.1371/journal.pone.0149533

**Published:** 2016-02-22

**Authors:** Yi Qiu, Shiwei Wang, Zhi Chen, Yajie Guo, Yuan Song

**Affiliations:** 1 Department of Microbiology, College of Biological Sciences, China Agricultural University, Beijing, PR China; 2 State Key Laboratory of Microbial Resources, Institute of Microbiology, Chinese Academy of Sciences, Beijing, PR China; Friedrich Schiller University, GERMANY

## Abstract

CRISPR-Cas systems, the small RNA-dependent immune systems, are widely distributed in prokaryotes. However, only a small proportion of CRISPR-Cas systems have been identified to be active in bacteria. In this work, a naturally active type I-E CRISPR-Cas system was found in *Streptomyces avermitilis*. The system shares many common genetic features with the type I-E system of *Escherichia coli*, and meanwhile shows unique characteristics. It not only degrades plasmid DNA with target protospacers, but also acquires new spacers from the target plasmid DNA. The naive features of spacer acquisition in the type I-E system of *S*. *avermitilis* were investigated and a completely conserved PAM 5’-AAG-3’ was identified. Spacer acquisition displayed differential strand bias upstream and downstream of the priming spacer, and irregular integrations of new spacers were observed. In addition, introduction of this system into host conferred phage resistance to some extent. This study will give new insights into adaptation mechanism of the type I-E systems in vivo, and meanwhile provide theoretical foundation for applying this system on the genetic modification of *S*. *avermitilis*.

## Introduction

Bacteria can be found nearly everywhere even in some harsh environments with the risk of predatory viruses and potentially harmful plasmids. In order to survive exposure to invasive genetic elements, bacteria have developed a variety of defense methods [1], including CRISPR-Cas systems, the small RNA-dependent immune systems [2]. CRISPR loci typically comprise clustered, noncontiguous direct repeats interspaced by variable sequences called spacers, and are frequently flanked by CRISPR-associated (*cas*) genes. CRISPR-Cas systems are widespread in bacteria and archaea, and are classified into three major types (type I, II and III) and 12 subtypes (I-A, I-B, etc.) according to the difference of *cas* gene contents and defense pathways across species [3].

Mechanisms of adaptive immunity mediated by CRISPR-Cas systems are intriguing and the subtype I-E CRISPR-Cas system in *E*. *coli* has been extensively studied [4, 5]. CRISPR loci in *E*. *coli* comprise multiple palindromic repeats of 29 nucleotides separated by variable spacers of 32 or 33 nucleotides [6]. Leader sequences flanking one side of each CRISPR locus appear to promote CRISPR transcription [7–9]. A cluster of eight *cas* genes (*cas3-cse1-cse2-cas7-cas5-cas6-cas1-cas2*) [2, 10] is adjacent to CRISPR I. Normally, the CRISPR-Cas system in *E*. *coli* is inactive because the expression of *cas* genes is repressed [2, 11]. However, when *cas1* and *cas2* are overexpressed with T7-*lac* promoter in *E*. *coli*, new spacers interspaced at the leader end of the arrays are obtained from intruding foreign genetic elements (called protospacers) [12, 13]. The selection of protospacers in invading nucleic acid usually depends on a proto-spacer-adjacent motif (PAM) and the typical PAM 5’-AAG-3’ has been identified in the system of *E*. *coli* [12–15]. Five Cas proteins, Cse1–Cse2–Cas7–Cas5–Cas6, referred to as CasABCDE, are involved in crRNA synthesis. In particular, a precursor RNA (pre-RNA) transcribed from a CRISPR array is processed into a mature CRISPR-RNA (crRNA) and then assembles with CasA_1_B_2_C_6_D_1_E_1_ to form the Cascade-crRNA complex [16]. Spacer acquisition from foreign nucleosides is strongly stimulated when crRNA matches protospacer (Referred to as priming). In addition to Cas1 and Cas2, the Cascade-crRNA complex ensures effective acquisitions through priming [12]. During the interference step, the crRNA is used as a guide for sequence-specific cleavage by Cas3 [2, 17].

*Streptomyces*, a genus of actinomycetes with a high G+C DNA content, are well known for their ability to produce many bioactive secondary metabolites [18]. Although many *Streptomyces* species encode CRISPR-Cas systems of similar structure, only one system has been characterized, and it is not able to provide immunity [19]. *Streptomyces avermitilis*, a producer of important pesticide avermectin, contains a CRISPR-Cas system that can be classified into the I-E subsystem [10].

However, the features and function of the type I-E system in *S*. *avermitilis* are unknown. Here, we describe the genetic characteristics of the CRISPR-Cas system in *S*. *avermitilis*. The natural adaptation and interference activity of this system was observed. In addition, this system was able to provide strain protection from the infection of phages with target protospacers. To our knowledge, it is the first time that the features of spacer acquisition in a naive subtype I-E system have been revealed.

## Materials and Methods

### Bacterial strains, phage and media

The wild-type strain *S*. *avermitilis* ATCC31267 (= MA-4680) and the high avermectin-producing strain 76–9 (derived from ATCC31267) were used [20]. Bacteriophages phiSASD1 and phiSAJS1 of *S*. *avermitilis* used in the phage assays were isolated and identified in our laboratory in the previous works [21]. YMS (0.4% yeast extract, 0.4% soluble starch, 1% malt extract, 0.0005% CoCl_2_⋅6H_2_O and 2% agar; pH 7.2) was used for sporulation or as a solid agar in the double layer assay. YEME (0.3% yeast extract, 0.5% peptone, 0.3% malt extract, 1% glucose and 25% sucrose) was used for liquid culture or as soft agar (0.7% agar) in the agar layer assay. EM (1% glucose, 0.4% tryptone, 1% yeast extract, 0.25% NaCl, 0.4% beef extract, and 2% agar) was used for the growth of mycelium and colony PCR. All *S*. *avermitilis* strains were incubated at 28°C. For the construction of plasmids and overexpression of *cas1-cas2*, *E*. *coli* JM109 and BL21 were respectively used.

### RNA isolation and RT-PCR

Total RNA was isolated from *S*. *avermitilis* using TRIZOL regent. Total RNA (2ug) was reverse transcribed into cDNA. The cDNA was amplified for 35 cycles for each primer pair. Meanwhile, RNA (2ug) was reverse transcribed similarly in the absence of reverse transcriptase and the product was amplified for 35 cycles for each primer pair to ensure there was no DNA contamination in the RNA samples or the reagents. In the semi-quantitative RT-PCR, primer pair (hrdB F and R) amplifying for RNA polymerase major sigma factor was used as an internal control to normalize the sample amounts.

### Plasmid loss and spacer acquisition

Oligonucleotides containing a 32-nt protospacer corresponding to CRISPR II spacer 16, with five upstream and downstream nucleotides, were synthesized (S1 Table) and cloned into pKC1139 (a multicopy vector containing the apramycin resistance gene *aac(3)Ⅳ*) [22] or pIJ653 (a multicopy vector containing the thiostrepton resistance gene *tsr*) using the restriction sites *Bgl*II and *Eco*RI to generate plasmid pKC1139-CRIIS16 (Apr^R^) or pIJ653-CRIIS16 (Thio^R^), respectively. The first base C of the protospacer was replaced with a T for plasmid pIJ653-CRIIS16CMT (Thio^R^). Oligonucleotides containing a 32-nt protospacer corresponding to CRISPR I spcer17 with an upstream 5’-AAG-3’ (S1 Table) were synthesized and cloned into pKC1139 using sites *Bgl*II and *Eco*RI to generate plasmid pKC1139-CRIS17 (Apr^R^). These plasmids were transformed into *S*. *avermitilis* protoplasts as previously described [23]. The transformants were selected and verified by colony PCR and then transferred to YMS agar containing apramycin (20 μg/ml) and incubated for 14–20 days. Spores were collected and spore suspensions were prepared as described in Practical *Streptomyces* Genetics [23]. Appropriate concentrations of the spore suspensions were spread on EM agar (for mycelia growth) without apramycin. Isolated colonies grown from spores were transferred to fresh EM agar with labeled squares for 3 days. Colony PCR was performed to detect plasmid loss using the primer pair aac F and R (S1 Table). Plasmid-free colonies were identified for spacer acquisition in CRISPR I or CRISPR II using the primer pairs CR I L F and R or CR II L F and R (S1 Table). For each colony, the same templates were used to detect plasmid loss and spacer acquisition. There were products of colony PCR using the primers CR I L F and R or CR II L F and R, and the results were the positive controls to confirm that the colony PCRs were working. Plasmid loss and spacer acquisition experiments were performed in triplicate for each strain with each plasmid. Spores from 3 plates were collected, and 144 colonies derived from spores of 3 plates were analyzed by colony PCR. Additionally, 400 other colonies of *S*. *avermitilis* ATCC31267 (containing pKC1139-CRIIS16) were screened for spacer acquisition. Spores were also assessed by replica plating technique. Appropriate concentrations of the spore suspensions were spread on EM agar without apramycin and grown for 3 days. Colonies were replica plated onto EM agar without apramycin, then immediately replicated onto apramycin-containing EM agar. More than 600 colonies without or with one plasmid were assessed.

If CRISPR arrays were inserted with new spacers, the products of colony PCR would show expanded bands with higher molecular weight in agarose gel electrophoresis. Expanded bands were subsequently sequenced. The acquired spacers were identified from the sequences by aligning them against wild-type CRISPR loci using DNAMAN V6. New spacers were aligned with the nucleotide sequences of the target plasmids (S2 Table). PAMs were identified by analyzing the upstream sequences of each protospacer using Weblogo Basic (http://weblogo.berkeley.edu/logo.cgi).

### Artificial CRISPR construction

Two protospacers downstream of the 5’-AAG-3’ PAMs from *holin* and *endolysin* genes of phiSASD1 were chosen for synthesis (S1 Table) and then ligated to the region from the leader to the 6^th^ repeat of CRISPR II using the restriction site *Bgl*II, which was designed as the 6^th^ spacer, according to the method described by Brouns et al [2]. The artificial CRISPR was inserted into multicopy vector pKC1139 (Apr^R^) or pSET152 (an integrative vector containing the apramycin resistance gene *aac(3)Ⅳ*) using *Bam*HI and *Xba*I. Plasmids were transformed into *S*. *avermitilis* 76–9.

### Plaque assays

A total volume of 100 μl phage lysates (10^3^ PFU/ml) was spread on the surface of YMS solid agar with 10 μg/ml apramycin. The soft agar of 5 ml of YEME contained 200 μl of an overnight culture of *S*. *avermitilis* 76–9 with plasmid. Following overnight culturing at 28°C, the numbers of plaques were counted. To plot the growth curve of phi SASD1, 1 ml cultures were collected from 25 ml YEME with 4×10^2^ PFU/ml phiSASD1 and 10^9^ cells/ml *S*. *avermitilis* 76–9 with plasmids every 24 h. One milliliter cultures were centrifuged immediately, and the PFUs of phiSASD1 in the supernatant were detected using the double layer assay. All of the plaque assays were performed in triplicate.

### Induction of spacer acquisition

The *cas1* and *cas2 genes* of *S*. *avermitilis* were cloned and ligated to the strong promoter pSD13 derived from *S*. *avermitilis* phage phiSASD1, which was identified in our previous study [24], using the restriction site *Bgl*II and then were inserted into the *E*. *coli-Streptomyces* shuttle plasmid pKC1139 using the restriction sites *Not*I and *Xba*I to generate the plasmid p13Cas1Cas2 (Apr^R^). Promoter pSD13 was ligated into pKC1139 to generate the control plasmid p13 (Apr^R^). It is noted that pSD13 promotes transcription without induction. Plasmid p13Cas1Cas2 was transformed into *E*. *coli* BL21 to detect the expression of *cas1* and *cas2*. BL21 cells containing plasmid p13Cas1Cas2 or plasmid p13 (control) were subcultured with a dilution of 1:100 into 25 ml LB with 100 μg/ml apramycin. Eight-milliliter cultures were separately collected after 5 and 11 h, washed with lysis buffer, centrifuged and then resuspended in 3 ml lysis buffer. Cells were lysed by ultrasonic treatment (200 W, 3 seconds each time, 50 times) and analyzed by SDS-PAGE on a 15% gel. Plasmid p13Cas1Cas2 (Apr^R^) was transformed into *S*. *avermitilis* 76–9. Spore suspensions were spread on EM agar without apramycin and screened for spacer acquisition by colony PCR. Plasmid pIJ653-CRIIS16 (Thio^R^) or pIJ653-CRIIS16CMT (Thio^R^) was transformed into protoplasts of *S*. *avermitilis* 76–9 (p13Cas1Cas2). The transformants harboring both plasmids (pIJ653-CRIIS16 or pIJ653-CRIIS16CMT and p13Cas1Cas2) were selected and then spores were collected. More than 100 colonies grown from spores were screened for spacer acquisition by colony PCR.

## Results

### The genetic characteristics of the subtype I-E CRISPR-Cas system in *S*. *avermitilis*

**Fig 1:**
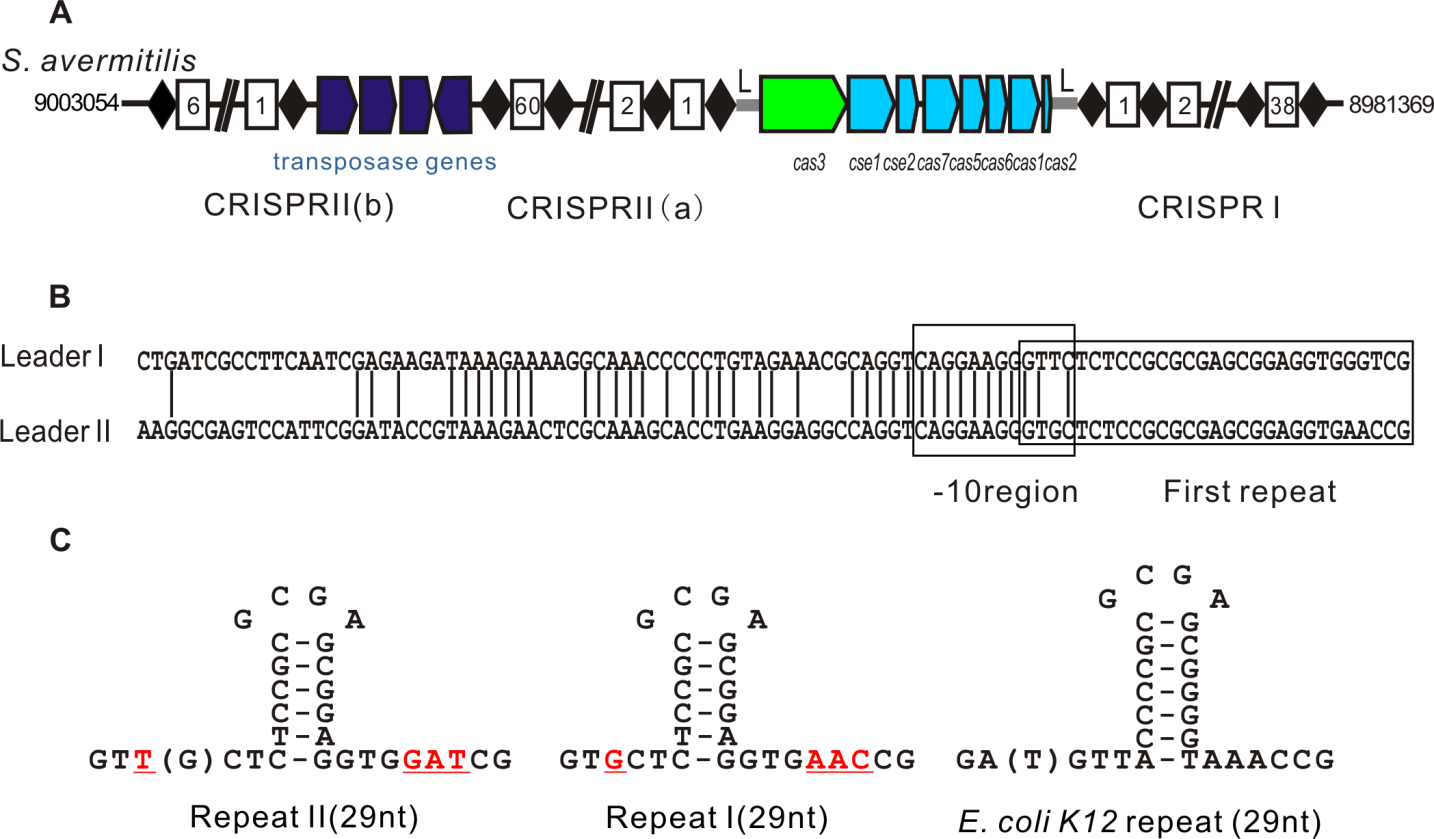
Genetic characteristics of the type I-E CRISPR-Cas system in *S*. *avermitilis* ATCC31267. (A) An overview of the CRISPR-Cas system locus in the *S*. *avermitilis* ATCC31267 genome. The eight *cas* genes are represented by arrows, and same-colored arrows indicate genes that are transcribed together. Repeats and spacers of CRISPR loci are represented by black diamonds and white rectangles, respectively. Numbers in white rectangles indicate the order of the spacers relative to the leader. The letter ‘L’ indicates the position of the leader. The four blue arrows represent the location of four transposase genes that separate the CRISPR II locus into two parts: CRISRP II (a) and CRISPR II (b). (B) The leader sequences of CRISPR I and CRISPR II are aligned. Straight lines indicate identical bases in the two leaders. The predicted -10 region is indicated in the gray box, and the first repeat is boxed. (C) Secondary structures of the repeats of CRISPR I and CRISPR II in *S*. *avermitilis* compared with those in *E*. *coli*. Repeat I or repeat II represents the repeat of CRISPR I or CRISPR II. The bases in the bracket represent changes in the sequences of minority repeats. Different bases between repeat I and repeat II are colored red and underlined. The sequence length of the repeats is indicated below.

The analysis of *S*. *avermitilis* ATCC31267 complete genome sequences (NC_003155.4) [25] using the CRISPI database (http://crispi.genouest.org/) [26] revealed a cluster of eight *cas* genes with two CRISPR loci, upstream CRISPR II and downstream CRISPR I. Moreover, the CRISPR II locus is interrupted by four transposase-encoding genes, dividing it into CRISPR II (a) and CRISPR II (b) (Fig 1A). Each CRISPR locus contains an A-T rich leader region adjacent to the first repeat. The -10 region of the leader was identified to overlap with the first repeat, using the promoter prediction database PRODORIC (http://prodoric.tu-bs.de/) (Fig 1B). The CRISPR I locus is transcribed from the same direction as the *cas* genes, whereas CRISPR II is transcribed from the opposite direction (Fig 1A). The sequences of the 29-bp repeats of the two CRISPR loci are different, but their secondary structures are similar to repeats found in *E*. *coli* (Fig 1C). The nucleotide sequences search of the 100 unique spacers using nucleotide BLAST (Basic Local Alignment Search Tool) (http://blast.ncbi.nlm.nih.gov/Blast.cgi) revealed that the CRISPR II spacer 16 showed 90% identity to plasmid pSTRVI01 (NC_015951.1) of *S*. *violaceusniger* Tu 4113. In addition, the corresponding protospacer in plasmid pSTRVI01 contains 5’-AAG-3’ upstream sequence, identical with PAM in *E*. *coli*. The CRISPR II spacer 18 shows 100% identity to the complete genomes of *S*. *davawensis* JCM 4913 (NC_020504.1; np: 5970920–5970951; gene product: multidrug resistance efflux protein) and *S*. *avermitilis* MA-4680 (NC_003155.4; np: 6243215–6243246; gene product: MFS transporter), but the corresponding protospacers lack the upstream 5’-AAG-3’. Other spacers had no homologs in the database.

The conserved domains of the eight Cas proteins showed strong homology with subtype I-E Cas proteins by protein BLAST (http://blast.ncbi.nlm.nih.gov/Blast.cgi) (S3 Table). Indeed, the CRISPR-Cas system of *S*. *avermitilis* has been classified as a subtype I-E CRISPR-Cas system [10]. Reverse transcriptase-polymerase chain reaction (RT-PCR) using specific primer pairs (S1 Table) revealed that eight *cas* genes and the CRISPR I and CRISPR II (a) loci are transcribed in vivo (S1A Fig). Moreover, *cse1-cse2-cas7-cas5-cas6-cas1-cas2* co-transcription was determined by RT-PCR with pairs of primers from adjacent *cas* genes (S1B Fig). The *cas3* gene is located in a separate operon from the other *cas* genes.

### Adaptation and interference activity of the type I-E system in *S*. *avermitilis*

To determine whether the type I-E system in *S*. *avermitilis* is active, we investigated its ability to eliminate target plasmids and acquire new spacers. Because it is difficult to analyze plasmid loss in individual *S*. *avermitilis* cells due to the mycelial development, individual spores were chosen as the units of analysis.

**Fig 2:**
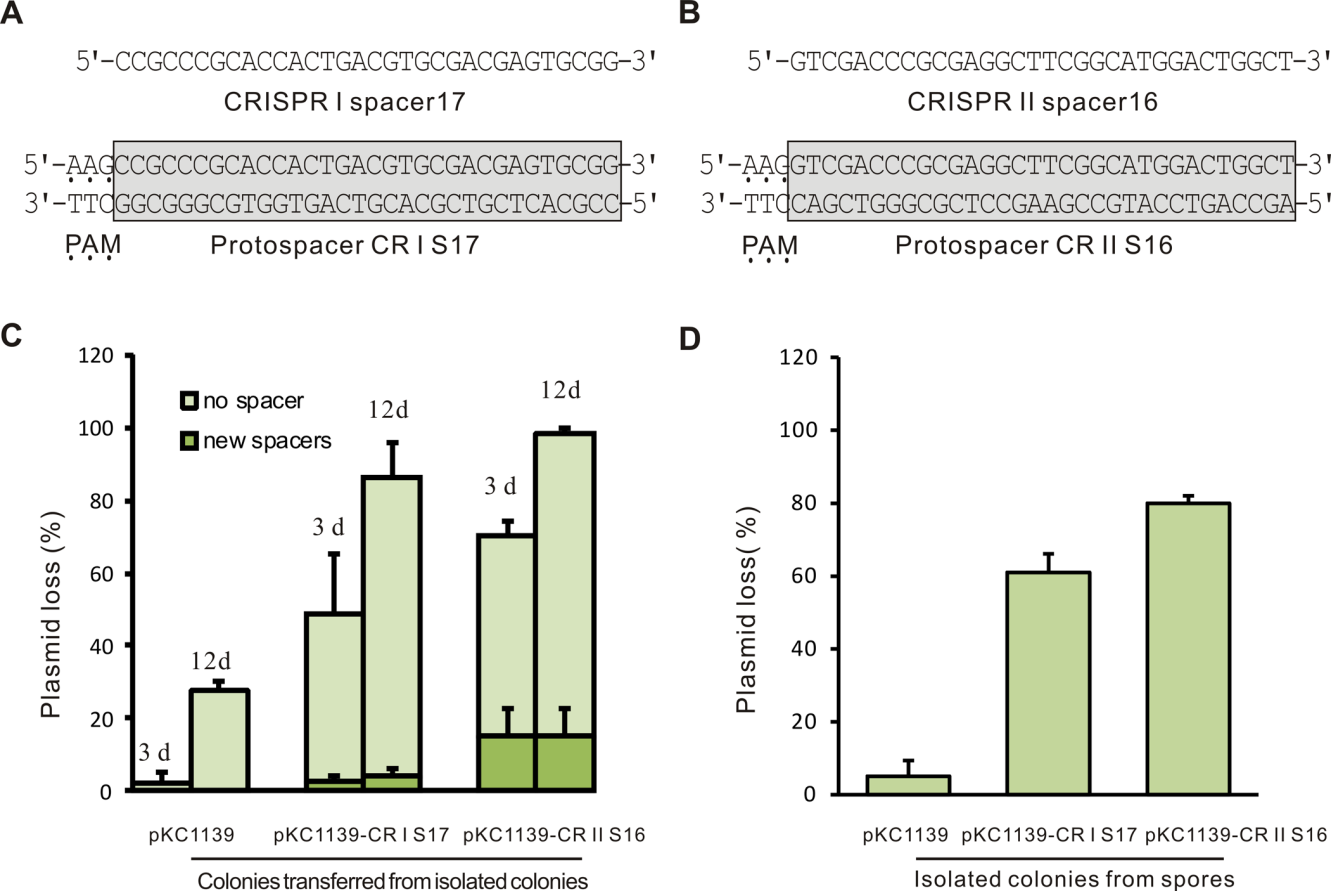
Adaptation and interference activity in the CRISPR-Cas system of *S*. *avermitilis*. (A) Protospacer CR I S17 (gray box) corresponding to CRISPR I spacer 17 with PAM upstream and (B) protospacer CR II S16 (gray box) corresponding to CRISPR II spacer 16 with PAM upstream were inserted into vectors. (C) Percentages of colonies that could not amplify the apramycin resistance gene by colony PCR. The labels ‘3 d’ or ‘12 d’ above the columns represent isolated colonies that were transferred to EM agar growing for 3 days or 12 days (transferred every 3 days for 4 times). The percentages of plasmid-free clones acquiring at least one new spacer (green) or no new spacer (light green) are shown. The derivation strains with plasmid pKC1139-CRIS17, pKC1139-CRIIS16 or empty vector pKC1139 are shown below. F tests showed significant differences in the percentages of colonies acquiring spacer between strain containing target plasmids and strain with empty vector (P<0.05). The percentage of colonies of strain containing pKC1139-CRIS17 or pKC1139-CRIIS16 without plasmids was significantly more than the percentage of colonies of strain with pKC1139 (P<0.05). (D) Percentages of isolated colonies from spores that could not grow on EM agar with apramycin. The derivation strains with plasmid pKC1139-CRIS17, pKC1139-CRIIS16 or empty vector pKC1139 are shown below. F tests showed significant differences in the percentage of colonies without plasmids between strain containing target plasmids and strain with empty vector (P<0.05).

Several plasmids, including the multicopy empty vector pKC1139, and two target plasmids (pKC1139-CRIS17 and pKC1139-CRIIS16 containing protospacers respectively corresponding to a spacer of CRISPR I and CRISPR II with an upstream 5’-AAG-3’), were transformed into *S*. *avermitilis* (Fig 2A and 2B). Isolated colonies from individual spores of *S*. *avermitilis* strains (containing plasmid pKC1139, pKC1139-CRIS17 or pKC1139-CRIIS16) were transferred to EM agar without apramycin. In order to detect plasmid loss and spacer acquisition during the colonies formation on agar without antibiotics, colony PCR was performed to detect plasmid loss of the sub-cultured colonies by amplifying *aac(3)IV*, the apramycin resistance gene. As shown in Fig 2C, 3% (4/144), 50% (71/144) and 70% (101/144) of the colonies of strains containing pKC1139, pKC1139-CRIS17 and pKC1139-CRIIS16 did not produce the expected sizes of PCR products. Spacer acquisition in plasmid-free colonies was analyzed by colony PCR amplifying the region containing the leader end of CRISPR arrays. The expanded bands on agarose gels indicated the CRISPR array with new spacers inserted in this region. DNA sequencing of expanded CRISPR arrays showed that 3% (4/144) and 15% (17/144) of colonies of strains containing pKC1139-CRIS17 and pKC1139-CRIIS16 acquired new spacers in the CRISPR I and CRISPR II loci, respectively. No new spacers were observed in the strain containing the empty vector pKC1139 (Fig 2C). Notably, when the CRISPR arrays of colonies were amplified, single expanded bands with higher molecular weight were observed instead of two bands comprising the parental band and the expanded band (S2 Fig). Sequencing of these expanded bands showed that all cells of a colony acquired the same spacer. It is unlikely that all cells of a colony would acquire the same spacer, unless spacer acquisition happened early in growth. These colonies were transferred from isolated colonies derived from individual spores. It is reasonable that new spacer was acquired in a unicellular spore, so all the cells of a colony derived from a spore have the same spacer. This finding indicated that many spacers were acquired in the spores. Another obvious question was whether the majority of spores lost the target plasmids. To detect plasmid loss in spores, we spread appropriate concentrations of the spore suspensions on EM agar without apramycin. Colonies were replica plated onto EM agar without apramycin, then immediately replicated onto apramycin-containing EM agar. The number of colonies without plasmids (failed to grow on EM agar with apramycin) was assessed. 5% (36/716), 61% (448/735) and 80% (507/634) of the colonies carrying pKC1139, pKC1139-CRIS17 and pKC1139-CRIIS16 lost plasmid, respectively (Fig 2D). This result, combined with the result detected by colony PCR, indicated that most plasmids lost during sporulation. More interestingly, even under the condition with antibiotic, spores from strains harboring pKC1139-CRIS17 or pKC1139-CRIIS16 exhibited high levels of plasmid loss. Summarily, new spacers could be acquired in both CRISPR loci, the CRISPR II locus displayed a stronger ability to eliminate plasmids and acquire spacers than the CRISPR I locus.

Previous studies demonstrated that more new spacers will be acquired in CRISPR loci after serial passaging [13]. To examine whether CRISPR-Cas system in *S*. *avermitilis* is the same situation, colonies harboring plasmids proved by colony PCR were transferred to fresh EM agar every 3 days for 3 times. Plasmid loss and spacer acquisition in colonies following sub-inoculations were detected. Nearly all of the strains with pKC1139-CRIS17 or pKC1139-CRIIS16 lost plasmids after 12 days, but only 2/93 colonies of strains with pKC1139-CRIS17 acquired new spacers after colonies were transferred for four times (Fig 2C). On contrast, 27% (39/144) of colonies of strains with an empty vector lost plasmids after 12 days. In conclusion, only a few acquisitions occurred during the growth of mycelia, indicating that new spacers may be acquired more frequently during the development of spores.

### Features of natural acquisition in the CRISPR-Cas system of *S*. *avermitilis*

**Fig 3:**
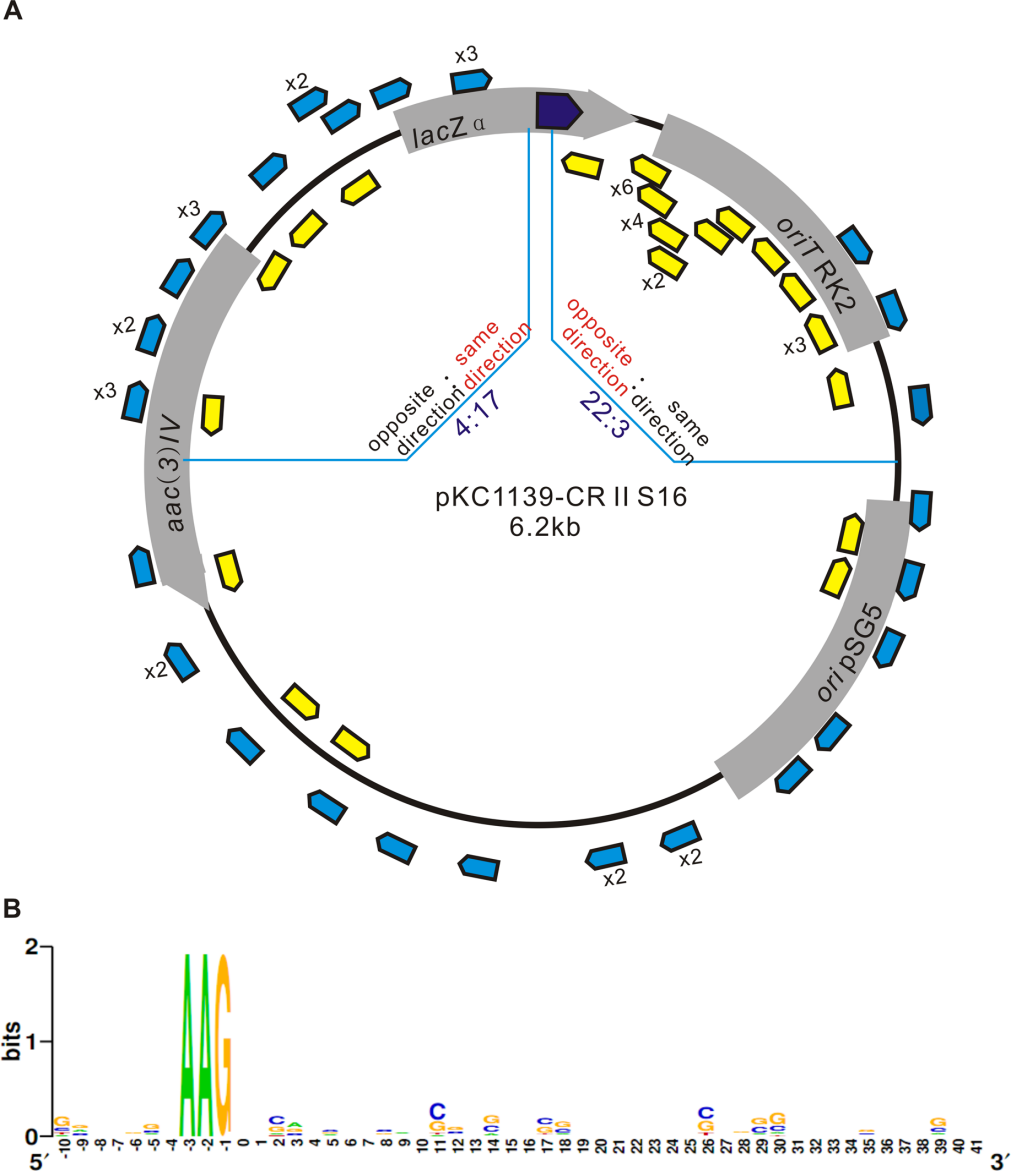
Orientation of protospacers and PAM motifs. (A) The dark blue arrow represents the location of the priming protospacer. Protospacers corresponding to acquired new spacers located on the plasmid DNA are indicated by blue arrows (protospacers derived from the same direction of the priming protospacer) and yellow arrows (protospacers derived from the opposite direction of the priming protospacer). Numbers next to arrows indicate the frequencies of identical spacers. The upstream and downstream regions near the priming protospacer are separated by light blue lines, and protospacers derived from two directions in the region are compared. (B) Ten nucleotides upstream and downstream of the protospacers were searched for PAM motifs using WebLogo. Position 0 represents the first nt of the protospacer.

To analyze spacer acquisition by the naive CRISPR-Cas system of *S*. *avermitilis*, another 400 colonies of strains carrying pKC1139-CRIIS16 were screened for new spacers. Due to the low frequency of natural spacer acquisition, only 69 new spacers (63 new spacers were acquired in strains with pKC1139-CRIIS16 and 6 in strains with pKC1139-CRIS17) and 50 unique spacers were obtained (S2 Table). All of the spacers were 32 bp in length. Among the spacers, 67 aligned with the target plasmids (Fig 3A). One new spacer was a duplication of the original first spacer and a CRISPR locus with the loss of the first repeat and spacer. No obvious strand bias for the orientation of protospacers was observed in *S*. *avermitilis*. However, downstream protospacers that approach the priming protospacer, with a ratio of 22:3, prefer to derive from the opposite direction of the priming protospacer (with respect to the PAM), whereas more upstream protospacers (17:4) tend to orient in the same direction of the priming protospacer (Fig 3A). Notably, differential strand bias upstream and downstream of the priming protospacer is also found in the naturally active type I-B system described in *Haloarcula hispanica* [27], but differs from the type I-E system found in genetically modified *E*. *coli*. In particular, in the type I-E system of *E*. *coli*, protospacers are derived from the same direction of the priming protospacer [12, 28]. Although the type I-B and type I-E systems both belong to the type I system, they differ with respect to their *cas* genes and CRISPR loci.

**Fig 4:**
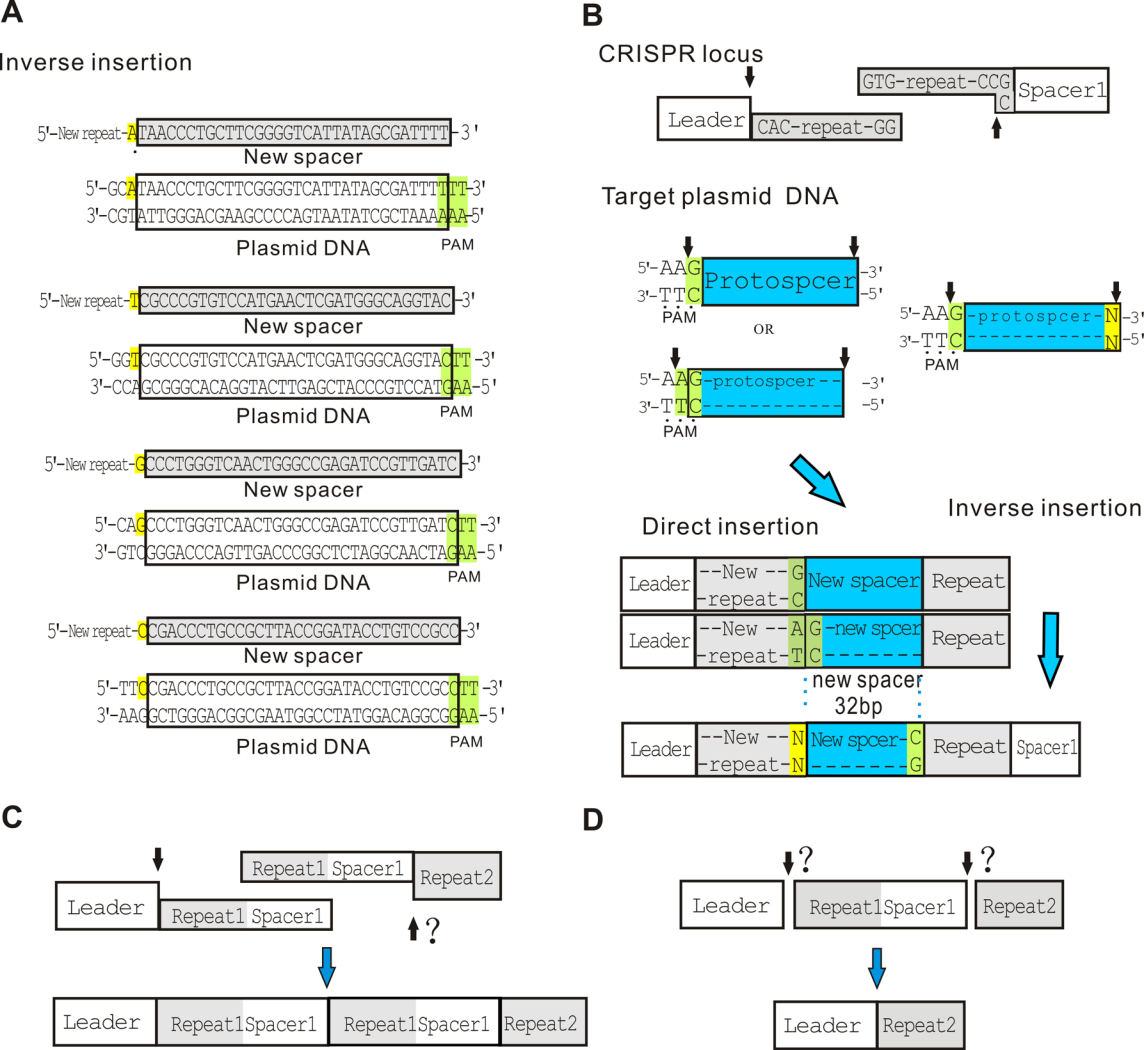
Irregular acquisitions observed in type I-E system in *S*. *avermitilis*. (A) Inverse insertion causes the differentiation of the last base of the new repeat. Protospacers from plasmid DNA and the corresponding new spacers are shown. The last base (yellow) of the new repeat corresponds to the first base (yellow) upstream of the protospacer. The PAM downstream of the protospacer is highlighted in green. (B) The proposed direct insertion and inverse insertion are shown. The leader and first spacer are represented by white rectangles. Repeat and protospacer are represented by a gray box and a blue box respectively. The black arrows indicate the proposed cleavage sites. Different instances of acquired fragments from target plasmid DNA are shown. The PAM upstream of the protospacer is underlined and the bases of PAM cleaved with protospacer are highlighted in green. The colors of inserted fragments from plasmid DNA are different from the original repeat sequences. New spacers are boxed, and sequence length is indicated below. (C) A CRSIPR II array with a new spacer that is a duplication of the original first spacer. The proposed cleavage sites are indicated black arrows. (D) A CRISPR II array showing loss of the first repeat and spacer. The possible cleavage sites are indicated with black arrows and question marks.

For all but four of the protospacers, the upstream PAM 5’-AAG-3’ was present, consistent with reported motifs from the type I-E system in *E*. *coli* (Fig 3B). The other four protospacers lacked an upstream 5’-AAG-3’ but had downstream 5’-TT-3’, and the last bases of these protospacers were C (T) (Fig 4A), indicating a 5’-CTT-3’ PAM. Considering the reported mechanisms of acquisition [12, 28–30], the irregular insertion manner could explain this result. The 5’-AAG-3’ motif is recognized and the last G base of the PAM along with the 32-bp protospacer is cleaved [12]. However, the 33-bp fragment inversely inserts into the CRISPR locus, and the last G base of the PAM becomes the last base of the new spacer (Fig 4B). This insertion manner is in contrast to direct insertion, in which the last G base of the PAM inserts along with the protospacer as the last base of the new repeat [12, 28]. Due to the inverse insertion, the last base of the new repeat changes with the first base upstream of the inversely inserted protospacer, as shown in Fig 4B. Moreover, during the cleavage of the protospacer, the last two A and G bases may sometimes be cleaved with the 31-bp protospacer, and the last base of the new repeat will therefore be A (Fig 4B). In addition to inverse insertions, there were other irregular acquisitions observed in the CRISPR-Cas system of wild *S*. *avermitilis*. For example, in one CRISPR II array, there was a duplication of the original first repeat and spacer, and it appears that the first spacer was cleaved and duplicated with the first repeat, although no new spacer was inserted into this CRISPR array (Fig 4C). In addition, the first repeat and spacer were deleted in one CRISPR II array, but it was unknown how to complete the excision (Fig 4D). Taken together, the naive CRISPR-Cas system of *S*. *avermitilis* appears to be quite reliable based on a unique PAM, although a small percentage of mistakes were observed.

### Inhibition of target phage expansion by the CRISPR-Cas system in *S*. *avermitilis*

**Fig 5:**
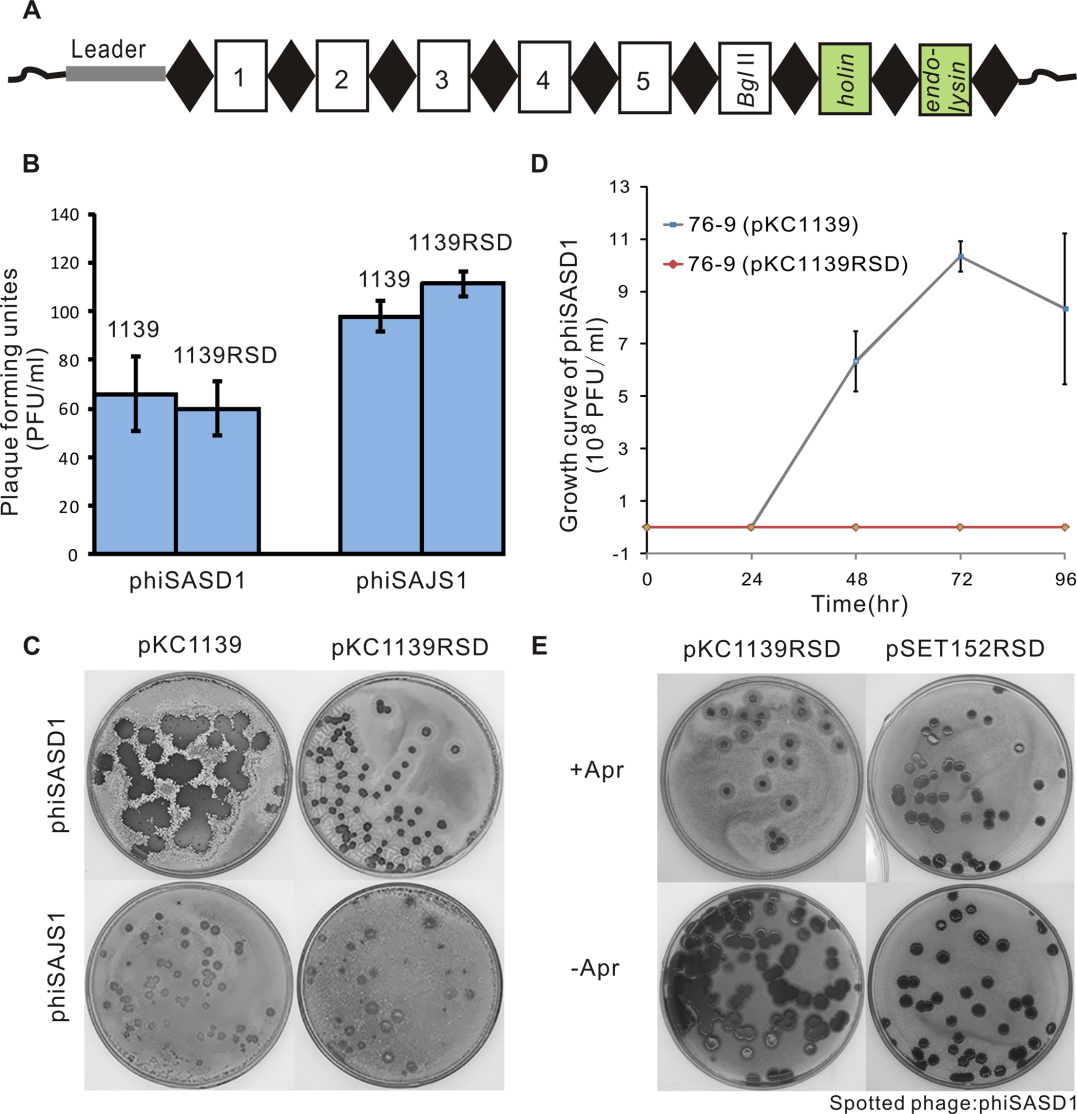
The CRISPR-Cas system in *S*. *avermitilis* provides protection from target phage infection. (A) A artificial CRISPR array with spacers targeting phiSASD1. Repeats and spacers of CRISPR loci are represented by diamonds and rectangles, respectively. Two spacers corresponding to *holin* and *endolysin* genes are colored green. (B) Plaque-forming units on *S*. *avermitilis* 76–9 containing empty vector or plasmid with artificial CRISPR array. *S*. *avermitilis* containing plasmids pKC1139 or pKC1139RSD as hosts are shown above. Tested phages phiSASD1 or phiSAJS1 are shown below. F test showed no significant difference in plaque-forming units between 76–9 containing empty vector and 76–9 containing the plasmid with artificial CRISPR array (P>0.05). (C) Plaques formed on *S*. *avermitilis* 76–9 containing pKC1139 or pKC1139RSD. Tested phages are indicated on the left. (D) Growth curves of phiSASD1 infecting *S*. *avermitilis* 76–9 with pKC1139 or pKC1139-RSD. The X-axis represents cultures of strains infected by phages that were collected every 24 h. The Y-axis represents the PFU/ml of lysates counted using the double layer technique (E) Plaques on *S*. *avermitilis* 76–9 with a multicopy vector (pKC1139-RSD) or an integrative vector (pSET152-RSD) with (+Apr) or without (-Apr) apramycin.

To determine whether the type I-E system in *S*. *avermitilis* can resist phage infection, an artificial CRISPR with two spacers targeting *S*. *avermitilis* phage phiSASD1 (NC_GQ379227) [21] was synthesized and cloned into vectors (Fig 5A). The *S*. *avermitilis* phage phiSAJS1 was used as a control and a high avermectin-producing strain derived from wild-type *S*. *avermitilis* 76–9 was used as a host in plaque-forming assays. Plaque-forming units of *S*. *avermitilis* 76–9 with the artificial CRISPR were compared with strains carrying an empty vector. As shown in Fig 5B, the CRISPR-Cas system could not prevent invasion by the phage phiSASD1 based on similar PFU counts in the two strains in the double layer assay. Remarkably, smaller plaques were formed on the lawns of strains with the artificial CRISPR than the strains with empty vector infected by the target phage phiSASD1, whereas similarly sized plaques were formed on the lawns of two strains infected by the non-targeted phiSAJS1 (Fig 5C), suggesting that plaque expansion was inhibited. Moreover, the explosion of small numbers of phages (approximately 400 PFU/ml) could be completely controlled in the strain carrying the artificial CRISPR, whereas the PFU of cultures of strains with the empty vector infected by phiSASD1 reached 10^8^ pfu/ml after 2 days (Fig 5D).

It was also observed that the resistance of multicopy plasmid pKC1139-RSD was stronger than the integrative vector pSET152-RSD (Fig 5E). The reason may be that pKC1139-RSD has higher transcription levels than pSET152-RSD. However, the integrative vector pSET152-RSD is more stable than the multicopy plasmid pKC1139-RSD. When apramycin was added in the solid agar of the double layer, both strains exhibited strong resistance, whereas the strains with pKC1139-RSD lost resistance when they were cultured on the plates without apramycin (Fig 5E). In conclusion, the CRISPR-Cas system in *S*. *avermitilis* can provide strains resistance against phage, and multicopy CRISPR arrays with functional spacers can increase phage-resistance.

### Low adaptation of the CRISPR-Cas system in *S*. *avermitilis*

Bacteriophage contamination occurs frequently during avermectin production, and a high-efficiency CRISPR-Cas system may be helpful for the construction of phage-resistant strains in industry. According to previous results, the CRISPR-Cas system in *S*. *avermitilis* exhibited weak ability to acquire new spacers. Cas1 and Cas2 play an important role in adaptation step [12–14]. To enhance spacer acquisition in this system, we constructed the *E*. *coli*-*Streptomyces* shuttle plasmid p13Cas1Cas2 that encodes Cas1 and Cas2 under the strong promoter pSD13 derived from the *S*. *avermitilis* phage phiSASD1 identified in our previous work [24]. The overexpression of Cas1 and Cas2 was confirmed in *E*. *coli* BL21 cells by SDS-PAGE (S3A Fig). Strong expression of Cas1 could be detected, whereas the MW of Cas2 was too small to be detected. Due to the co-transcription of the two genes, we believe that they were co-expressed at similar levels. We also detected the overexpression of Cas1 and Cas2 at the transcriptional level by semi-quantitative RT-PCR (S3B Fig). However, no new spacer was identified in the overexpression strains.

Because the PAM and seed sequences of the protospacer with mismatches may stimulate the acquisition of new spacers, defined as the priming process [14], protospacer CR II S16CMT, whose first C base was replaced with a T, was inserted into plasmid pIJ653. The plasmid pIJ653-CRIIS16CMT was transformed into the strain overexpressing Cas1 and Cas2. However, no new spacers were observed in the strain with pIJ653-CRIIS16CMT and p13Cas1Cas2, even though this mismatch can cause priming, as shown in *E*. *coli* [14]. However, one 33-bp and two 32-bp new spacers were identified in the strain harboring pIJ653-CRIIS16 and p13Cas1Cas2. A BLAST search indicated that the spacers were all derived from the genomic DNA of *S*. *avermitilis* (np: 4047768–4047799, membrane protein; np: 5586770–5586802, type VII secretion-associated serine protease; np: 8556310–8556341, polyketide synthase). As the three nucleotides upstream of each protospacer were not 5’-AAG-3’, self-targeting was avoided. Taking together, the failure of activation of adaptation may suggest that the system in *S*. *avermitilis* is different from that in *E*. *coli*.

## Discussion

In recent years, intense investigations have been performed to reveal the molecular mechanisms of the type I-E CRISPR-Cas system in *E*. *coli*. However, activity of this system is repressed in wild-type *E*. *coli*, hindering studies of the inherent function of type I-E CRISPR-Cas systems in this species. In this work, we describe a naturally active type I-E CRISPR-Cas system in *S*. *avermitilis*. Although this system possesses similarities to the type I-E system in *E*. *coli*, it shows some unique characteristics, particularly with respect to spacer acquisition.

Adaptation is the most intriguing step of CRISPR-Cas system. There are two stages of adaptation, “naive adaptation” and “primed adaptation” [31]. In the naive adaptation stage, spacers are acquired from new foreign genetic elements. In the primed adaptation stage, additional spacers are acquired from the invading cognate DNA. In our study, a target plasmid was introduced into the strain, which could lead to degradation. However, the results showed that new spacers were acquired from the target plasmid with the completely complementary PAM and protospacer (Fig 2C), which was quite unexpected. Recent studies have indicated that foreign DNA with the target PAM-protospacer should be directly degraded, and the intruding DNA with the mutation on the PAM or seed region can promote spacer acquisition [12, 14]. It has been proposed that in the type I-E system of *E*. *coli*, point mutations in the PAM or protospacer weaken the interaction of the Cascade-crRNA complex with the protospacer [32]. This decreased interaction abolishes direct degradation of the target DNA and triggers the acquisition of new spacers from foreign DNA [14, 31]. Therefore, primed adaptation is a positive response to intruders that escape direct interference through mutation of their PAM or protospacer. However, it is unclear how plasmid with the target PAM-protospacer promotes primed adaptation in *S*. *avermitilis*, and further investigation is needed. Furthermore, we observed spacer acquisition occurred in spores grown under antibiotic pressure. Without antibiotic, cells do not keep foreign plasmids. However, under the pressure of antibiotic, cells are faced with the choice of keeping antibiotic-resistant plasmids or targeting potentially harmful plasmids by CRISPR-Cas systems. Ultimately, it appears that most cells choose to lose their plasmids, and some of these cells acquire new spacers to destroy the plasmids more effectively. However, cells without plasmids must then face damage due to antibiotics. Therefore, CRISPR-Cas systems are not always beneficial. Indeed, it has been proposed that active CRISPR-Cas systems hamper bacteria from acquiring beneficial genes encoding antibiotics or virulence factors from foreign genetic elements. By contrast, some bacteria choose to keep these beneficial genes instead of activating the CRISPR-Cas systems [5].

In *S*. *avermitilis*, we observed that spacer acquisition and plasmid loss were more common during sporulation than aerial mycelium formation. This may be due to the complex morphological differentiation of *Streptomyces*. The aerial hyphae develop into chains of spores that contain many tens of genomes and then subdivide into unigenomic spores. During genome replication in the spore chain, the CRISPR-Cas system is naturally multiplied. Therefore, it appears that the activity of the CRISPR-Cas system is enhanced during sporulation. Many actinomycetes develop mycelia and spores. We analyzed the published genomic sequences of actinomycetes using the CRISPI database and found that many actinomycetes contain CRISPR-Cas systems. Three types of systems have been found in actinomycetes, and most can be classified into subtype I-E systems (Table 1). Furthermore, a single strain can contain two subtype systems and multiple CRISPR arrays. For most of the type I-E systems in actinomycetes, two CRISPR arrays are transcribed from opposite directions. Based on the characteristics of the type I-E systems in *S*. *avermitilis*, CRISPR loci downstream of the *cas* genes transcribe from the same direction as the *cas* genes, whereas CRISPR arrays upstream may transcribe from the opposite direction. The repeat sequences of different species are unique, but the bases CC(T)CCGCNNNNGCGGG(A)G forming the palindromic structure of the repeats are conserved, implying that type I-E systems in different genera of actinomycetes function in a similar way and may be active (Table 1).

**Table 1:** Many types of CRISPR-Cas systems have been found in the genus actinomycetes.

Genus	Most types in this genus^a^	Representative strain	Repeat sequence^b^
*Bifidobacterium*	Type I-A	*B*. *adolescentis* ATCC 15703 (NC_008618)	GTCGCTCTCCTTACGGAGAGCGTGGATTGAAAT
	Type I-E	*B*. *animalis subsp*. *lactis* ATCC 27673 (NC_022523)	GTGTTCCCCGCAAGCGCGGGGATGATCCC
*Kitasatospora*	Type I-E	*K*. *setae* KM-6054 (NC_016109)	CTCGGCCCCGCGCTCGCGGGGGTTGCTC
*Streptomyces*	Type I-E	*S*. *avermitilis* MA-4680 (NC_003155)	GTGCTCTCCGCGCGAGCGGAGGTGAACCG
*Nocardiopsis*	Type I-E	*N*. *dassonvillei subsp*. *dassonvillei* DSM 43111 (NC_014210)	GTGCTCCCCGCGCACGCGGGGATGGTCCC
*Acidothermus cellulolyticus*	Type II	*Acidothermus cellulolyticus* 11B (NC_008578)	CCATTTTAGCCGGGGGATTGAGACAGGCTC CCCAGC
*Frankia*	Type I-C	*Frankia sp*. CcI3 (NC_007777)	GCAGCGCCGGGCGTCCGCGCCCGGCGAGGTTCCCAAC
	Type I-E		GTCGTCCCCGCACGCGCGGGGATCTTCC
*Propionibacterium*	Type I-E	*P*. *acidipropionici* ATCC 4875 (NC_019395)	GTCGTCCCCGCGCAGGCGGGGGTAATCCG
*Amycolatopsis*	Type I-E	*A*. *mediterranei* RB (NC_022116)	GGGACCAGCCCCGCGCGTGCGGGGACAAC
*Saccharomonospora*	Type I-E	*S*. *viridis* DSM 43017 (NC_013159)	GTCCGCCCCGCGCATGCGGGGATGAACCG
*Corynebacterium*	Type I-E	*C*. *aurimucosum* ATCC 700975 (NC_012590)	GTGCTCCCCGCGTAAGCGGGGATGAGCCC
*Nocardia*	Type I-E	*N*. *farcinica* IFM 10152 (NC_006361)	GTGCTCCCCGCGCGTGCGGGGATGAGCCC
*Mycobacterium*	Type III-A	*M*. *africanum* GM041182 (NC_015758)	GTCGTCAGACCCAAAACCCCGAGAGGGGACGGAAAC

^a^ Type I-E CRISPR-Cas systems are highlighted in gray.

^b^ Palindromic structures of repeats of type I-E CRISPR-Cas systems are underlined.

We have demonstrated that the native CRISPR-Cas system in *S*. *avermitilis* provides host resistance to phage to some extent, while type II CRISPR-Cas system-based phage-resistant *S*. *thermophiles* mutants have already been applied successfully in the food industry [33]. The type I-E system in *S*. *avermitilis* possesses strong activity during inference step, although it exhibits weak ability to acquire new spacers. Furthermore, naive adaptation and primed adaptation induced by mutant plasmids have not been detected in *S*. *avermitilis*. In the CRISPR-Cas system of *E*. *coli*, strong adaptive ability can be obtained by overexpressing *cas1* and *cas2*. However, overexpression of *cas1* and *cas2* in *S*. *avermitilis* did not increase adaptation and instead led to the nonspecific recognition of genomic DNA. Weak adaptation hampers the application of this system in the construction of CRISPR-mediated phage resistant strains. In addition, *S*. *avermitilis* strains have been used in the industrial production of avermectin for many years, and it was found that many strains have lost the native CRISPR-Cas systems due to subculture (data not show). One possible reason for this is that the CRISPR loci of *S*. *avermitilis* are on the right arm of the chromosome, a region that is inherently unstable, and undergoes deletion [34]. Therefore, further efforts are need to explore the construction of CRISPR-mediated phage-resistant strains in industry.

Members of the genus *Streptomyces* play critical roles in the production of various beneficial secondary metabolites in industry, and many genes are involved in the synthesis of secondary metabolites. Modification of these genes could improve the production secondary metabolites, and efficient gene editing tools are needed. Recently, an engineered CRISPR-Cas9 system has been successfully applied in *Streptomyces* for genome editing, and the invention of sgRNAs and the combined CRISPR/Cas9-CodA(sm) system has further simplified this technology [35, 36]. Type I systems composed of multiple protein subunits may not be a suitable tool for genetic engineering [37]. However, it is possible to employ native *cas* genes of the type I-E system to perform genome editing. In *S*. *avermitilis*, we have observed several strains that could not eliminate target plasmids, even after several passages. These plasmids were observed to have a DNA fragment deletion within the PAM and protospacer. If the type I-E system in *S*.*avermitilis* can make double-strand DNA breaks at target sites by transforming into a synthetic CRISPR array with spacers matching the target DNA, specific mutations may occur. Double-strand breaks can promote homologous recombination, when cells are introduced exogenous DNA fragments homologous to the cleaved region. According to the proposed mechanism of Cas3 action, it is possible that the Cascade–crRNA complex and Cas3 protein can generate a double-strand DNA breaks [17]. However, Cas3 may degrade genomic DNA prior to homology-directed repair (HDR) or nonhomologous end joining (NHEJ) [37]. Therefore, a proper target site may be able to avoid genome degradation. Further experiments are needed to explore the applications of this system in genome editing. Many sequenced *Streptomyces* species harbor active CRISPR-Cas systems such as the one found in *S*. *avermitilis*, as shown in Table 2. It will be useful to study the features and activity of CRISRP-Cas systems in other *Streptomyces* species.

**Table 2:** *Streptomyces* strains and *Streptomyces* plasmids harbor type I-E CRISPR-Cas systems.

Genus	Strains	Repeat sequence
*Streptomyces*	*S*. *avermitilis* MA-4680 (NC_003155)	GTGCTCTCCGCGCGAGCGGAGGTGAACCG
	*S*. *sp*. Mg1 (NZ_CP011664)	GTGCTCTCCGCGCGAGCGGAGGTGGGTCG
	*S*. *nodosus* ATCC 14899 (NZ_CP009313)	GTCCTCTCCGCGCGAGCGGAGGTGAGTCG
	*S*. *kanamyceticus* NBRC 13414 (AB254080)	GTGCTCTCCGCGCGAGCGGAGGTGGGTCG
	*S*. *bottropensis* ATCC 25435 (NZ_KB911581)	GTGCTCTCCGCGCGAGCGGAGGTGATCCG
	*S*. *albus* DSM 41398 (NZ_CP010519)	GTGCGCTCCGCGCGAGCGGAGGTGAGCCG
	*S*. *albus* J1074 (NC_020990)	CTGCTCCCCGCGCGTGCGGGGTTGGTCCC
	*S*. *ghanaensis* ATCC 14672 (NZ_DS999641)	GTCCTCCCCGCCGATGCGGGGGTGTTCCG
	*S*. *xiamenensis* 318 (NZ_CP009922)	GTGGTCCCCGCACACGCGGGGATGGTCCCC
	*S*. *lydicus* A02 (CP007699)	GTCGTCCCCGCACCCGCGGGGGTTGTCC
	*S*. *ambofaciens* ATCC 23877 (CP012382)	CTGCTCCCCGCACCCGCGGGGATGGTCCC
	*S*. *albulus* NK660 (NZ_CP007574)	CCGCTCCCCGCACCCGCGGGGATGAGCCC
	*S*. *vietnamensis* GIM4.0001 (CP010407)	CTGCTCCCCGCACCCGCGGGGATGGTCCC
	*S*. *sp*. *CFMR 7* (NZ_CP011522)	GTCCTCCCCGCCGACGCGGGGGTGTTCCG
	*S*. *sp*. CNQ-509 (NZ_CP011492)	CTGCTCCCCGCGTACGCGGGGATGGACCC
	*S*. *davawensis* JCM 4913 (NC_020504)	GTGCTCCCCGCACCCGCGGGGATGGTCCC
	*S*. *tsukubaensis* NRRL18488 (JX081647)	GTGCTCCCCGCACGCGCGGGGATGGTCCC
	*S*. *griseus subsp*. griseus NBRC 13350 (NC_010572)	GTGGTCCCCGCGCGTGCGGGGTTGTTCCC
*Streptomyces* plasmids	*S*. *sp*. HK1 plasmid pSHK1 (NC_010311)	GTCGGCCCCGCACCCGCGGGGATGCTCC
	*S*. *violaceusniger* Tu 4113 plasmid pSTRVI01 (NC_015951)	GTGCTCTCCGCGCGAGCGGAGGTGAGCCG
	*S*. *sp*. CFMR 7 plasmid (NZ_CP011523)	GTGCTCTCCGCGCGAGCGGAGGTGAGCCG
	*S*. *pratensis* ATCC 33331 plasmid pSFLA01 (NC_016110)	GTGCTCCCCGCGCGTGCGGGGATGGTCCC
	*S*. *hygroscopicus subsp*. jinggangensis TL01 plasmid pSHJGH1 (NC_020894)	GTGCTCCCCGCGCCCGCGGGGATGGTCCC
	*S*. *sp*. PAMC26508 plasmid pSP01 (NC_021056)	GTGCTCCCCGCGCGTGCGGGGATGGTCCC

In conclusion, we described an active type I-E system in *S*. *avermitilis*. This study highlights the inherent function of the type I-E system in *S*. *avermitilis*. We observed novel features of type I-E system during the spacer acquisition, which could give new insights into adaptation mechanism of CRISPR-Cas systems. This system was shown to protect strains from infection by target phage and may be used to construct phage-resistant strains.

## Supporting Information

S1 Fig Transcription and co-transcription of *cas* genes in *S*. *avermitilis*. (PDF)

S2 Fig Expanded bands from amplified CRISPR arrays. (PDF)

S3 Fig The overexpression of Cas1 and Cas2 was detected. (PDF)

S1 Table Primers and synthesized oligonucleotides used in this study. (DOCX)

S2 Table New spacers aligned with targeted DNA. (DOCX)

S3 Table The conserved domains of the eight Cas proteins were analyzed by protein blast. (DOCX)
