# Supplementary material for: An Active Type I-E CRISPR-Cas System Identified in Streptomyces avermitilis
Source: PLoS One. 2016 Feb 22;11(2):e0149533. doi: 10.1371/journal.pone.0149533 (PMC4762764; doi:10.1371/journal.pone.0149533)
Supplement: S1 Fig — (PDF) [file pone.0149533.s001.pdf]

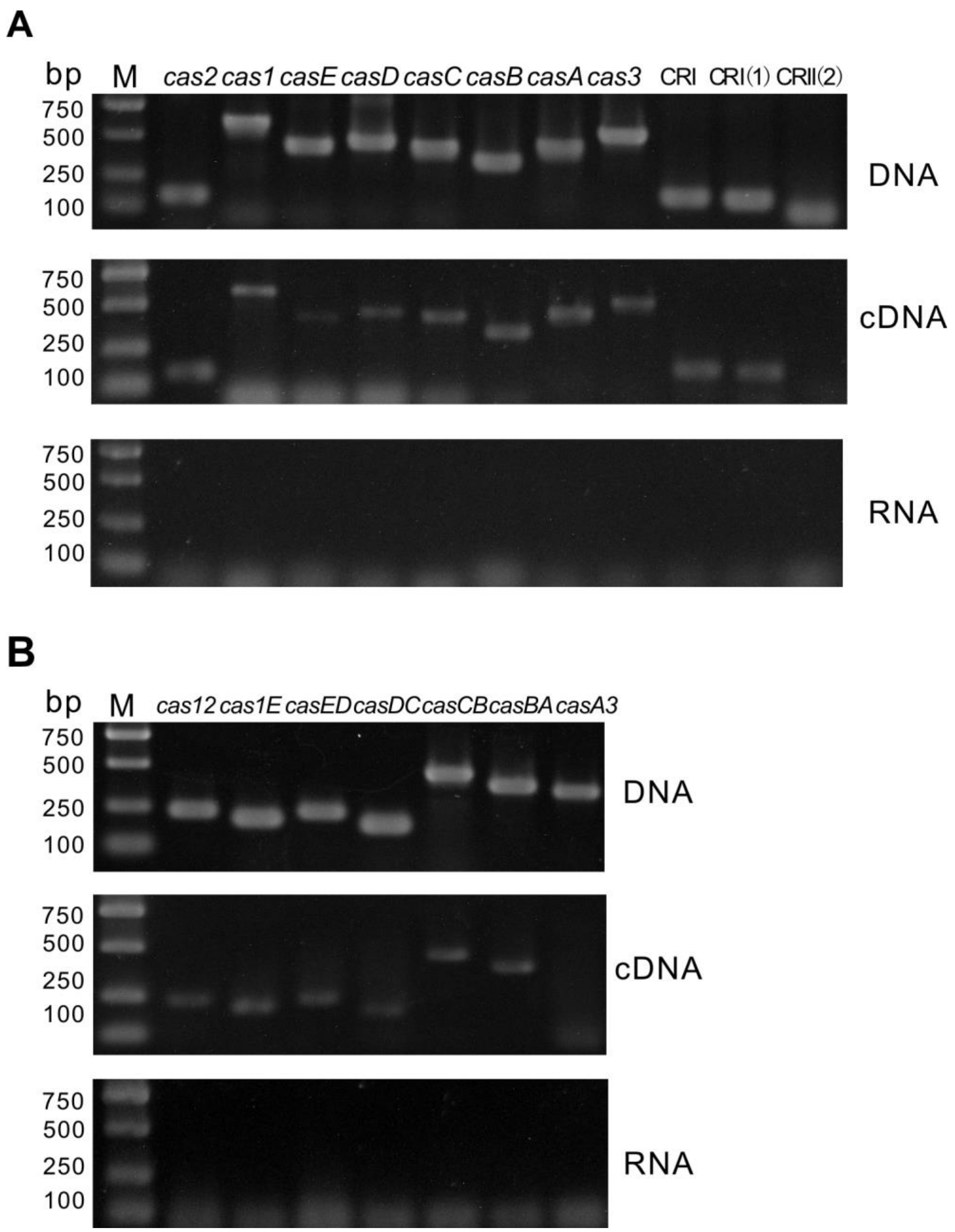

S1 Fig. Transcription and co-transcription of *cas* genes in *S. avermitilis*. (A) RT-PCR revealed that eight *cas* genes, as well as CRISPR I and CRISPR II (a) are transcribed in vivo. (B) Co-transcription of *cas* A-B-C-D-E-1-2 was verified by RT-PCR. In (A) and (B), DNA template, cDNA template or RNA template of *S. avermitilis* used in PCR amplification is shown right. DNA markers are shown left.
