# Supplementary material for: An Active Type I-E CRISPR-Cas System Identified in Streptomyces avermitilis
Source: PLoS One. 2016 Feb 22;11(2):e0149533. doi: 10.1371/journal.pone.0149533 (PMC4762764; doi:10.1371/journal.pone.0149533)
Supplement: S2 Fig — (PDF) [file pone.0149533.s002.pdf]

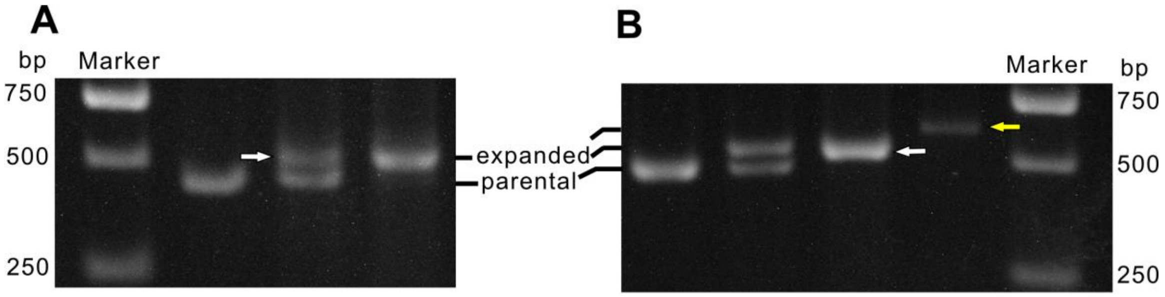

S2 Fig. Expanded bands from amplified CRISPR arrays. The parental band and expanded band of CRISPR I (A) or CRISPR II (B) are shown on agarose gels. Two bands in the second lane represent the parental and expanded bands. The white arrows indicate the position of expanded bands containing one new spacer. The expanded band containing two new spacers is indicated by a yellow arrow.
