# Supplementary material for: An Active Type I-E CRISPR-Cas System Identified in Streptomyces avermitilis
Source: PLoS One. 2016 Feb 22;11(2):e0149533. doi: 10.1371/journal.pone.0149533 (PMC4762764; doi:10.1371/journal.pone.0149533)
Supplement: S3 Fig — (PDF) [file pone.0149533.s003.pdf]

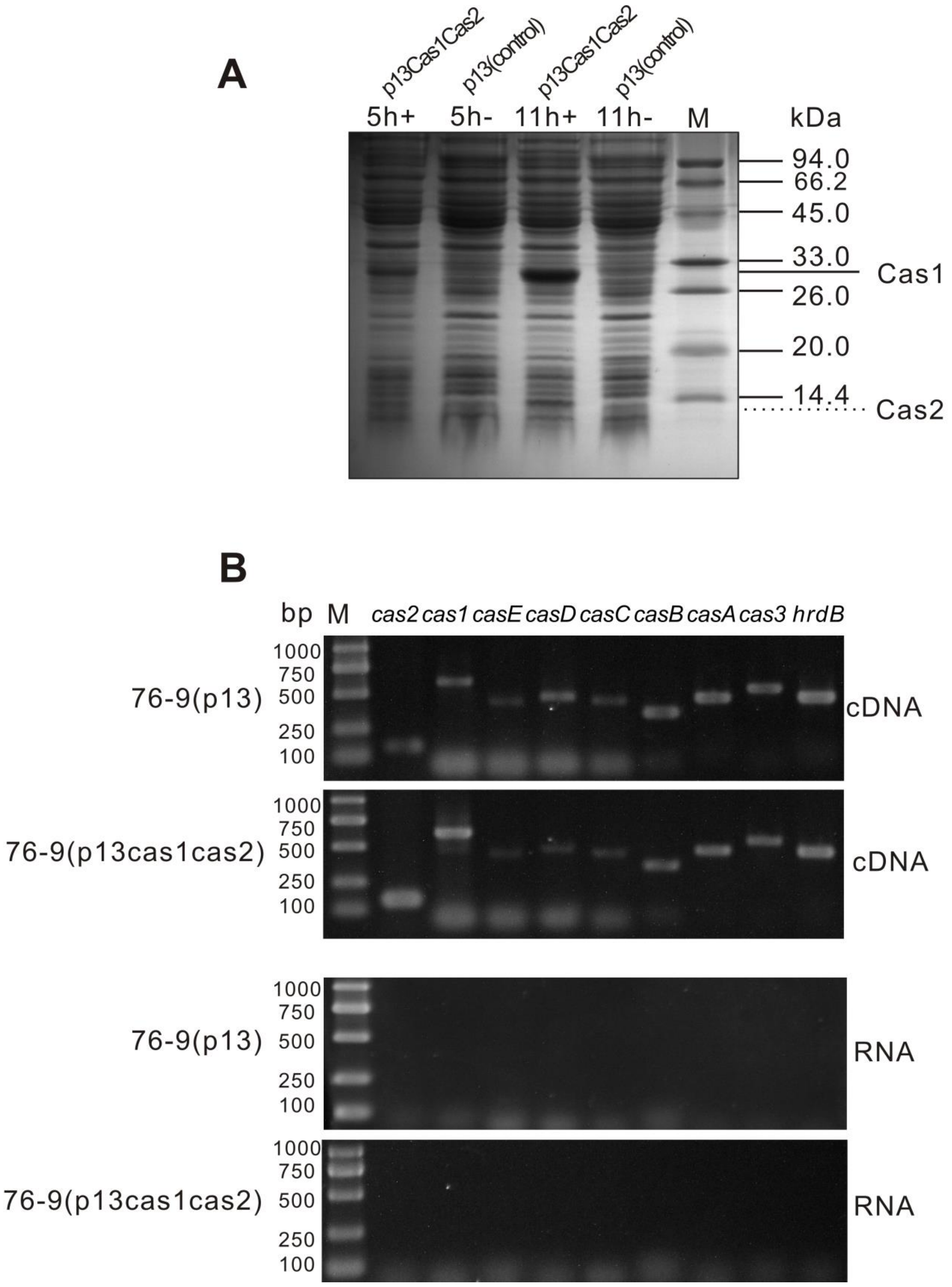

S3 Fig. The overexpression of Cas1 and Cas2 was detected. (A) The overexpression of Cas1 and Cas2 in *E. coli* BL21 cells was analyzed by SDS-PAGE. BL21 harboring p13Cas1Cas2 or p13 (control) was cultured for 5 or 11 h. Cultures were analyzed by

SDS-PAGE on a 15% gel. The black arrows indicate the expected size of Cas1 (32.9 kDa) and Cas2 (10.8 kDa). The expression level of Cas2 could not be determined. The marker is shown on the left. (B) The transcription of *cas1* and *cas2* was detected by semi-quantitative RT-PCR. cDNA templates or RNA templates used for PCR amplification and DNA markers are shown right. cDNA templates of 76-9(p13Cas1Cas2) or 76-9(p13) are shown left. Primer pairs amplifying for *cas* genes are shown above and *hrdB* was used as an internal control to normalize the sample amounts.
