# Supplementary material for: An Active Type I-E CRISPR-Cas System Identified in Streptomyces avermitilis
Source: PLoS One. 2016 Feb 22;11(2):e0149533. doi: 10.1371/journal.pone.0149533 (PMC4762764; doi:10.1371/journal.pone.0149533)
Supplement: S1 Table — (DOCX) [file pone.0149533.s004.docx]

**S1 Table. Primers and synthesized oligonucleotides used in this study.**

| **Name^a^** | **Sequence(5'-3')^b^** | **Description** | **Restriction**  **site** |
| --- | --- | --- | --- |
| CR1-F | TGCCGTGGACGTTGGT | CRISPRI transcription detection |  |
| CR1-R | GCGTTGCTCGGTGGTT |  |  |
| CR2-F | GCGGGTCGGGTTGTAGT | CRISPRII(A) transcription detection |  |
| CR2-R | GGCGATGCTGCTGAT |  |  |
| CR3-F | GCTCACCGTCGTGCTCT | CRISPRII(B) transcription detection |  |
| CR3-R | CCCGCACCTCGTTGTC |  |  |
| cas0001-F | CAGTGCGGAGCTCGAAG | *cas0001* transcription detection |  |
| cas0001-R | CACGCTGGTTGTTGGAAG |  |  |
| cas1-F | GCCGATTCGCTGTCCTTCCT | *cas1* transcription detection |  |
| cas1-R | CCTGTTGGTTGCCGTTGTGG |  |  |
| casE-F | CTCTTCCGCATCGAGGACAC | *casE* transcription detection |  |
| casE-R | TGCCTTCGGTGATCTTCTGG |  |  |
| casD-F | ACGCCGTCTTCACCATCGC | *casD* transcription detection |  |
| casD-R | GCTCCCTTCTGGGTGGTTGAGAT |  |  |
| casC-F | CGGCGTCATCAACCTCTTC | *casC* transcription detection |  |
| casC-R | TCGGTGCGGACGGAAAG |  |  |
| casB-F | CAGAGCGTCACCGAGAATCCA | *casB* transcription detection |  |
| casB-R | TCACGGCGAGTGCGAAGG |  |  |

**S1 Table. Continued.**

| **Name** | **Sequence(5'-3')** | **Description** | **Restriction site** |
| --- | --- | --- | --- |
| casA-F | CGCTCCTTCGATCTGTGCC | *casA* transcription detection |  |
| casA-R | GCCTTCTGCTCTGTGAAACCC |  |  |
| cas3-F | GGTCCTCAAAGCCAAAGAA | *cas3* transcription detection |  |
| cas3-R | GGTCGGAAGCGCAAAA |  |  |
| cas10001-F | AACAGCTCGTCGATCTATG | *cas1 and cas0001* co-transcription detection |  |
| cas10001-R | ACCGCAGTCCATAATTCG |  |  |
| cas1E-F | ACGATGTCCAGGTAGAGG | *cas1 and casE* co-transcription  detection |  |
| cas1E-R | CCAGAAGATCACCGAAGG |  |  |
| casED-F | TCCTCGATGCGGAAGAG | *casE and casD* co-transcription  detection |  |
| casED-R | ACAGACCACCTCACCAG |  |  |
| casCD-F | CGTTCGTTGAAGTGGCTG | *casC and casD* co-transcription  detection |  |
| casCD-R | TGGAGACCAAGGACCTCA |  |  |
| casCB-F | GTTGAGGTTGGCGAAGG | *casC and casB* co-transcription  detection |  |
| casCB-R | CGGCTGAATCTGCTGAC |  |  |
| casBA-F | GGCGGTAGTTGGATTCTC | *casB and casA* co-transcription  detection |  |
| casBA-R | GCCGAGTACATGAACGA |  |  |
| casA3-F | GCATCGGACTCCTATCG | *casA and cas3* co-transcription  detection |  |
| casA3-R | TGCTGCGTGATCTGGT |  |  |

**S1 Table. Continued.**

| **Name** | **Sequence(5'-3')** | **Description** | **Restriction**  **site** |
| --- | --- | --- | --- |
| hrdB-F | GCTCTTCCTGGACCTCAT | amplifying for RNA polymerase major sigma factor |  |
| hrdB-R | GTACACCTTGCCGATCTC |  |  |
| aac-F | ACAGGCAGAGCAGATCAT | amplifying for *aac*(3)Ⅳ |  |
| aac-R | ATCGCATTCTTCGCATCC |  |  |
| CR I L-F | AAGCGGTAGCATTCACG | amplification for new spacer on CR I |  |
| CR I L-R | GAAACGCAGGTCAGGAAGG |  |  |
| CR II L-F | GCGACCTTCTCACGCATTT | amplification for new spacer on CR II |  |
| CR II L-R | CACGATACTCATGCTCACA |  |  |
| p13-F | AGCGGCCGCTGTACGGCGCTGATTACTGA | amplification for promoter p13 | *Not*I |
| p13-R | GAAGATCTGACAGACATGGAGGTCTCCTCT |  | *Bgl*II |
| Cas1Cas0001-F | GAAGATCTGTGAACACTGCAGCCACACC | amplification for *cas1cas0001* | *Bgl*II |
| Cas1Cas0001-R | GCTCTAGACCTTCCTGACCTGCGTTTCT |  | *Xba*I |
| Leader-F | CGGGATCCCAACGCATCCTCACCTG | amplification for leader region of  CRISPR II | *Bam*HI |
| Leader-R | GAAGATCTGATCATTGCGTCTTGAGTTA |  | *Bgl*II |

**S1 Table. Continued.**

| **Name** | **Sequence(5'-3')** | **Description** | **Restriction**  **site** |
| --- | --- | --- | --- |
| CR I S17 | CGGAATTCAAGGTCGACCCGCGAGGCTTCGGCATGGACTGGCTAGATCTTCGGATCCCG | oligonucleotides corresponding to CRISPRI spacr18 | *Eco*RI  *Bgl*II |
| CR II S16 | CGGAATTCTCAAGCCGCCCGCACCACTGACGTGCGACGAGTGCGGCTGGCAGATCTTC | oligonucleotides corresponding to CRISPRII spacr16 | *Eco*RI  *Bgl*II |
| CR II S16CMT | CGGAATTCAAGTCGCCCGCACCACTGACGTGCGACGAGTGCGGAGATCTTCGGATCCCG | oligonucleotides corresponding to CRISPRII spacr16(The first base C of protospacer was replaced by T ) | *Eco*RI  *Bgl*II |
| RSD | GAAGATCTGG AGTGCTCTCC GCGCGAGCGG AGGTGAACCC GGGCCCTTGC CCGACTTCTG  GGGCATGATC ACGTGCTCTC  CGCGCGAGCG GAGGTGAACC  CCCGCGACCG TTCGTCGCCA  GGGCGTTGAC TTGGTGCTCT CCGCGCGAGC GGAGGTGAAC CCAAGCTTGG G | synthesizing artificial CRISPR | *Bgl*II  *Hind*Ⅲ |

^a^ F or R indicates forward or reverse primer.

^b^ Restriction endonuclease sites are underlined.
