# Supplementary material for: An Active Type I-E CRISPR-Cas System Identified in Streptomyces avermitilis
Source: PLoS One. 2016 Feb 22;11(2):e0149533. doi: 10.1371/journal.pone.0149533 (PMC4762764; doi:10.1371/journal.pone.0149533)
Supplement: S2 Table — (DOCX) [file pone.0149533.s005.docx]

**S2 Table. New spacers aligned with targeted DNA.**

| New spacers acquired from plasmid pKC1139-CR II S16^a^ | | | | | |
| --- | --- | --- | --- | --- | --- |
| **Spacer No.^b^** | **New spacer** | **Target nucleotides** | **Strand** | **PAM** | **Last base**  **of new repeat** |
| A1 | TGGCCCATCTTCGAGGGGCCGGACGCTACGGA | 4923-4954 | - | AAG | G |
| A2 | GCTGACCGATGAGCTCGGCTTTTCGCCATTCGT | 4838-4869 | - | AAG | G |
| A3 | GTCCGGTGTAACGGGCGACGTGGCAGGATCGA | 5043-5074 | - | AAG | G |
| A4 | GTCGCGCTGATTGCTGGGGCAACACGTGGAGC | 5648-5679 | + | AAG | G |
| A5 | ACGATAGTTACCGGATAAGGCGCAGCGGTCGG | 4288-4319 | - | AAG | G |
| A7.2 | CCGAGGGTCTGCCTGCCGTGAGGTGGCCGGCG | 175-206 | - | AAG | G |
| A8 | GCAAGACCGATCCCCGGGGACCTGCATAGATC | 3522-3553 | + | AAG | G |
| A9 | CCAACCAGGAAGGGCAGCCCACCTATCAAGGT | 3083-3052 | + | AAG | G |
| A10 | CGGGCAGTGAGCGCAACGCAATTAATGTGAGT | 3716-3747 | - | AAG | G |
| A11 | CCCCGCCCGGGCCTGGGCGGGGCTTCCTGTGC | 2337-2368 | + | AAG | G |
| A12 | CCGAGGGTCTGCCTGCCGTGAGGTGGCCGGCG | 175-206 | - | AAG | G |
| A13 | TCTACACGAACCCTTTGGCAAAATCCTGTATA | 2830-2861 | - | AAG | G |
| A14 | GCGGACAGGTATCCGGTAAGCGGCAGGGTCGG | 4143-4174 | - | AAG | G |
| A15 | GTCCGGTGTAACGGGCGACGTGGCAGGATCGA | 5043-5074 | - | AAG | G |
| A16 | ACCAAGGGCGGCGTCGCCAAGATCGGCCTGGA | 1067-1098 | - | AAG | G |
| A17 | AAGAGCGACTTCGCGGAGCTGGTGAAGTACAT | 3476-3507 | + | AAG | G |
| A18 | GCGGCAGGGTCGGAACAGGAGAGCGCACGAGG | 4124-4155 | - | AAG | A |
| A19 | CCCGCGTCCGGATTACCCGCTGTAGTCGTGCT | 1934-1965 | + | AAG | G |
| A20 | ACACGACTTATCGCCACTGGCAGCAGCCACTG | 4340-4371 | + | AAG | G |

**S2 Table. Continued.**

| **Spacer No.** | **New spacer** | **Target nucleotides** | **Strand** | **PAM** | **Last base**  **of new repeat** |
| --- | --- | --- | --- | --- | --- |
| A21 | TGCAATGTACGTACGCTGGTTTCCCTCCAGAA | 5998-6029 | + | AAG | G |
| B1 | CACGTCCCCATGCGCTCCATCAAGAAGAGCGA | 3452-3483 | + | AAG | G |
| B2 | GCAAGACCGATCCCCGGGGACCTGCATAGATC | 3522-3553 | + | AAG | G |
| B3 | CGGGCAGTGAGCGCAACGCAATTAATGTGAGT | 3716-3747 | - | AAG | G |
| B4 | TACATCACCGACGAGCAAGGCAAGACCGATCC | 3503-3534 | + | AAG | G |
| B5 | TTTATCACCACCGACTATTTGCAACAGTGCC | 4751-4782 | + | AAG | G |
| B6 | TCGTCGCGCATCGTGCGCCAGCGGCGGCCGGA | 1424-1455 | + | AAG | G |
| B7 | CTCGCCCCGGTACTCCAGCCCTGGGGCCAGCA | 323-354 | - | AAG | G |
| B8 | CCGGGCGAGGCGACGTGGGGCAACACGTGCCG | 1686-1717 | - | AAG | G |
| B9 | CTGACCGATGAGCTCGGCTTTTCGCCATTCGT | 4839-4870 | - | AAG | G |
| C1 | GCAAGACCGATCCCCGGGGACCTGCATAGATC | 3522-3553 | + | AAG | G |
| C2 | CACGTCCCCATGCGCTCCATCAAGAAGAGCGA | 3452-3483 | + | AAG | G |
| C3 | TGGCGCAACGTGTTCGTGCCAAGGTGCAGCCC | 1550-1581 | + | AAG | G |
| C4 | GGCGCGTACGGCTCGTCCGGCCGCCGCTGGCG | 1439-1470 | - | AAG | G |
| C5 | TACATCACCGACGAGCAAGGCAAGACCGATCC | 3503-3534 | + | AAG | G |
| D1 | ACAATCCCCGATCCGCTCCACGTGTTGCCCCA | 5661-5692 | - | AAG | G |
| D2.1 | GTTGAGAAGCCGCAGAATCCCTTTCTGGAGGGA | 6018-6049 | - | AAG | A |
| D2.2 | ACCGATCCCCGGGGACCTGCATAGATCTGCCA | 3528-3559 | - | AAG | G |
| D3 | CGGGCAGTGAGCGCAACGCAATTAATGTGAGT | 3716-3747 | - | AAG | G |
| D4 | ATGCAGCGTCGTGTTGGCATCGTGTCCCACGC | 5261-5292 | - | AAG | G |

**S2 Table. Continued.**

| **Spacer No.** | **New spacer** | **Target nucleotides** | **Strand** | **PAM** | **Last base**  **of new repeat** |
| --- | --- | --- | --- | --- | --- |
| D5 | GCAAGACCGATCCCCGGGGACCTGCATAGATC | 3522-3553 | + | AAG | G |
| D6 | GAACTCGGCATCCAGCAGGACGTCCGCACCGA | 839-870 | - | AAG | G |
| D7 | TACATCACCGACGAGCAAGGCAAGACCGATCC | 3503-3534 | + | AAG | G |
| D8 | GCAAGACCGATCCCCGGGGACCTGCATAGATC | 3522-3553 | + | AAG | G |
| D9.1 | GCGGACAGGTATCCGGTAAGCGGCAGGGTCGG | 4143-4174 | - | AAG | G |
| D9.2 | CGGCAGGGTCGGAACAGGAGAGCGCACGAGGG | 4123-4154 | - | AAG | G |
| D10 | CCCCGCCCGGGCCTGGGCGGGGCTTCCTGTGC | 2338-2369 | + | AAG | G |
| D11 | GCAAGACCGATCCCCGGGGACCTGCATAGATC | 3522-3553 | + | AAG | G |
| D12.1 | CACTACCGGTCGCAGAACCTGAACCGGCTGAT | 875-906 | - | AAG | G |
| D13 | AATGCGATGCCGCTCGCCAGTCGATTGGCTGA | 5576-5607 | + | AAG | G |
| D14 | TACCTGCCCATCGAGTTCATGGACACGGGCGA | 5219-5250 | - | AAG | G |
| D15 | TACCTGCCCATCGAGTTCATGGACACGGGCGA | 5218-5249 | - | AAG | G |
| D16 | CCCCGCCCGGGCCTGGGCGGGGCTTCCTGTGC | 2337-2368 | + | AAG | G |
| D17 | CAGGACGAGCTTGGCAAGGTCATGATGGGCGT | 3349-3380 | + | AAG | G |
| D18 | GACCAGCCTCCTCACATGAGTAGGCTGAAACA | 6079-6110 | - | AAG | G |
| D19 | ATCAACGGATCTCGGCCCAGTTGACCCAGGGC | 5512-5543 | - | AAG | G |
| D20.1 | TCCTGGGTTGTGTTCGCCTTGGTAGCGCTCCT | 6162-6193 | - | AAG | G |
| D20.2 | TACCTGCCCATCGAGTTCATGGACACGGGCGA | 5219-5250 | - | AAG | G |

| **S2 Table. Continued.**   \| **Spacer No.** \| **New spacer** \| \| **Target nucleotides** \| \| **Strand** \| **PAM** \| **Last base**  **of new repeat** \| \| \| --- \| --- \| --- \| --- \| --- \| --- \| --- \| --- \| --- \| \| Exceptional spacers^c^ \| \| \| \| \| \| \| \| \| \| D16 \| Deletion of the first spacer and repeat \| \| \| \| \| \| \| \| \| A6 \| ACACGGCTGTTGAGCCGGACGGCGCCGAGACC \| Same with the original first spacer \| \| \| \| \| \| \| \| A7.1 \| CGCCCGTGTCCATGAACTCGATGGGCAGGTAC \| 5220-5251 \| \| + \| \| TT downstream \| \| T \| \| C6 \| CCCTGGGTCAACTGGGCCGAGATCCGTTGATC \| 5513-5544 \| \| + \| \| TT downstream \| \| G \| \| C7 \| TAACCCTGCTTCGGGGTCATTATAGCGATTTT \| 2770-2801 \| \| + \| \| TT downstream \| \| A \| \| D12.2 \| CGACCCTGCCGCTTACCGGATACCTGTCCGCC \| 4144-4175 \| \| + \| \| TT downstream \| \| C \|  \| New spacers acquired from plasmid pKC1139-CR I S17^d^ \| \| \| \| \| \| \| --- \| --- \| --- \| --- \| --- \| --- \| \| **Spacer No.** \| **New spacer** \| **Target nucleotides** \| **Strand** \| **PAM** \| **Last base**  **of new repeat** \| \| A1 \| CTCGCCCCGGTACTCCAGCCCTGGGGCCAGCA \| 323-354 \| - \| AAG \| G \| \| A2 \| TACATCACCGACGAGCAAGGCAAGACCGATCC \| 3534-3503 \| + \| AAG \| G \| \| B1 \| GGAAAATCTCCGTCCACTGCAGGTCAGAGCGC \| 1889-1920 \| - \| AAG \| G \| \| B2 \| CTGACCGATGAGCTCGGCTTTTCGCCATTCGT \| 4832-4863 \| - \| AAG \| G \| \| B3 \| CCCCGCCCAGGCCCGGGCGGGGCTTCTTCCGA \| 2327-2358 \| - \| AAG \| G \| \| C1 \| ATCGAAGAGAAGCAGGACGAGCTTGGCAAGGT \| 3337-3368 \| + \| AAG \| G \| |
| --- | --- | --- | --- | --- | --- | --- | --- | --- | --- | --- | --- | --- | --- | --- | --- | --- | --- | --- | --- | --- | --- | --- | --- | --- | --- | --- | --- | --- | --- | --- | --- | --- | --- | --- | --- | --- | --- | --- | --- | --- | --- | --- | --- | --- | --- | --- | --- | --- | --- | --- | --- | --- | --- | --- | --- | --- | --- | --- | --- | --- | --- | --- | --- | --- | --- | --- | --- | --- | --- | --- | --- | --- | --- | --- | --- | --- | --- | --- | --- | --- | --- | --- | --- | --- | --- | --- | --- | --- | --- | --- | --- | --- | --- | --- | --- | --- | --- | --- | --- | --- | --- | --- | --- | --- | --- | --- | --- | --- | --- | --- | --- | --- | --- | --- | --- | --- | --- | --- | --- | --- |

**S2 Table. Continued.**

| New spacers acquired from genome DNA^e^ | | | | | | |
| --- | --- | --- | --- | --- | --- | --- |
| **Spacer No.** | **New spacer** | **Target nucleotides** | **Strand** | **Three nucleotides upstream**  **of protospacer** | **Last base**  **of new repeat** | **Spacer length(nt）** |
| 1 | CGCAAGCGTCGCCGTGCTCAGGCGACATCACAT | 5586770-5586802  type VII secretion-associated serine protease | - | GTC | C | 33 |
| 2.1 | CCGGCTGTTCGCCCCGCTCCTGGGCCGCTCCA | 8556310-8556341  polyketide synthase | - | CGA | A | 32 |
| 2.2 | CAGCGAGCTGACGACCGAGCCGCCCGCCTTGT | 4047768-4047799  [membrane protein](http://www.ncbi.nlm.nih.gov/nucleotide/162960844?report=gbwithparts&from=4047510&to=4048451&RID=PWXK6Y08015) | + | GCG | G | 32 |

^a d e^ Derivation DNA of new spacers are indicated.

^b^ A, B and C correspond to new spacers screened from triplicate experiments of spacer acquisition. D represents spacers screened from other 400 colonies from *S.avermitilis* ATCC 31267 (pKC1139-CR II S16). The followed number represents the colony number. For some colonies, two new spacers are acquired. The spacer near the leader is represented by 1 separated by dot with colony number, and 2 represents new spacer acquired firstly.

^c^ New spacers corresponding to exceptional protospacers of which PAM are not 5’-AAG-3’ are highlighted yellow.

**SUPPLEMENTARY SEQUENCE**

**Nucleotide sequences of pKC139-CR II S16**

1

CACCGGTCCGTCGACCACCA CCAGGACGTCGTTGTCGACG TCGGCCCCGCGGCTCCTGCC CGCCGAACGCGTCGTCGTCG ACGGCCTGGTGCTCATCGAC

GAGCACCCGGAGCCAGGTGA AAAGCGCCGGCGGACGCTCG GACTGGGCGCGGGATTCCAG CAGTAACCCAGGTCCGCCGG CCACCTCACGGCAGGCAGAC

CCTCGGCTTTCGCGCCGGGA CCGCCATGAGACCGCCACCC GGATGTCCGGGGTGGCGGTC TCATGGCGGTCCCTCAGCGG CCCTACGCGACCGCTGTGTC

GACGCGGAGGCAGTCTCCGG GGTGCTGGCCCCAGGGCTGG AGTACCGGGGCGAGCTTGCC CTTGCAGCGGCGGCACACGG GCGACGCGGCGCCGGGCGGG

GGAGTCTTGACGTCGACGGT GACCGGCTTCGGCTTCAGGC GCTTCCGAAGGCTGATCGTC GGGCGGATCGCCTCCTTCTT CGGCTGACGCACTCGGTCGA

GCGAAGCGGCCAGCTCCTCC GTGCGGGCCTCGTTGGCCTT CCGGCGCGCCTCGCGGAAAG CAGCTTCCTCCTCGGACATG ACCTCGAACCTCATCTGGTC

AGCGTCAAGGTCGCCCGGCG CGGCCGGCGCTTCCGGGGCG GGCGGGTCCAGGACGTCCTT GCCCCACACCAGGCCCCAGG ACTCGACGAGCCGCCGGACG

CCCGGTAGGCCGTACGTCTC GGCGACCTTGATGAGGTCGA GGCGACGTCCGGCGACGCGG GCGATGTATCGGTACCAGAT GTAGGCCGGGATGACCGCGA

TGGCGACCAGGCCCTCGGTG TCGTCGGTGATCTCCTCCTC GGTGCGGACGTCCTGCTGGA TGCCGAGTTCCTTGATCAGC CGGTTCAGGTTCTGCGACCG

GTAGTGCTTGCGGACCTGGA AGACGCCGAACTCGCGCTCG CGGTACTTCTCGACGAACGG GCCGGGCCGACGAAGCCGCT GCAGCTCGGCGGCCGCCGCG

TCGCCCAGGTCGAGCGGTCC CATGCGGTCGTCGCCGCGAC CGGCCTTGAAGTTCTGTCCG GCCAGCTCCAGGCCGATCTT GGCGACGCCGCCCTTGGTCT

TGTCGCCGTCCTTGTAGAGG TAGCGGGCCTGCTTGCCCGC ATCGCCGTCAGCGGCGTCCG CGCCGTTGAGTGGGCGCACG TCGGTGCCGTGGCCCTTGCC

CTCACAGGAGCAACCGGGCC GGTCGCACGTCTCGCTGACG GTGTAGCCGCCCGCGGATTC GACCCCGGCGGCCCAGGCTC CGGCGAGTGCGTCGCGGAAC

GCGGCCTGGGCGTCCGGGCC GAGCACCTCGCGGGTGACCC AGAGCGTGTGCCAGTGCAGG TGCCAGCCGGAGCCCCAGCC GAAGGTGTCCTCGAAGGCCC

GCTCGTAGCCGATGATCCCG AAGTCGTCGCGCATCGTGCG CCAGCGGCGGCCGGACGAGC CGTACGCGCCCTTCCAGCCG TCGTGCAAGACCGCGACCAG

GCCGTGCCGCATTCCCTTGC GGACGGTGCCGAACGCCATG CGCTCGAAGTGGCGCAACGT GTTCGTGCCAAGGTGCAGCC CGTACCCGGCGTCCGCGAGA

CCGTCGGCGGCGAGCTGCAC GTTCGAGCCCCGTACGGCCA GGATGCGGCTCATGCACCAC GGGCAGGTGTGGACGTTGTT GCAGCGGCACGTGTTGCCCC

ACGTCGCCTCGCCCGGCTTC CACATCAGCTCGGCCGTCCC GGCAGTGAGCCGGGTCCCGC AGCCCTTGAACGCCTCGTTC AGCGACACCGTCTGGTGCCG

GTCCCGCCGGGCGAACCGCT CGTCGCGCGGGTCCTCCCGC CCGGCTGTCGCGGCACCCTC GTTTGGGGTAGAACCCGTTC CAGTTACAGCGCTCTGACCT

GCAGTGGACGGAGATTTTCC CTTACTACTAAAGCCCGCGT CCGGATTACCCGCTGTAGTC GTGCTTGCTACGCTGCGTGA CTGGTCCGCAATGAGACGCT

TTGCGCGCTTTCGGCAGGCG TCCGAGCAGTAGATTTTGGG GCGCTTCCCGGGGATGTGGA CGATCGGGGTGCCGCAGTGG CACTTCGGTCCGGCGGGGCG

CGGTGGTGTCGACGCGCTGT TCTCTCGTACGCTCGTCACA GAGCAAACGTCCTCACTCGG CATGCTGCGCCGGTTCGGGG GCGGCGAGCCCGGGAGGCCA

ATCCCGGGCTCGTGCCATTT CTGGGTCCTGTTGATCCTGG CATTGGTGTGGCCGTTCATT GCCCCTGCTCGCTCCTGACG CGCCGATAGACGTCCGATAC

GCCCGGTGCTGGTGGGATTT GATAGGTCGGAAGAAGCCCC GCCCGGGCCTGGGCGGGGCT TCCTGTGCGTCAGGACCTCC TCGTCGTGAGCCTCTTCGGC

CTATGGACGGAGTGACCTCG TGATCCGTTACAGCCGCGCG CGCTCGCGTAGAGCGGTCTC ATCAGTTCCACGAACGGTCC TCTTCGCAGATCAGGGCGTT

GGGGCGGAGTCTCACCAAGG ACTACGTCTGCTGGCGATTT CCGTTACACCCCGGGCGGTG GCCGGCGCACACGCGCGCCC GCGTTGGGCAGTGCAGAAAG

TGCAGAAACCTAGGCGCTGA TGGTCCAGGTCCACGGTTCG TCGTCGGCGGCGGCGCGGGC GGCGGCGTCGGCCAGGGCGC GGGCGAGACCGGCTACGGCG

GGCTTGATGCGCCGGTTGCG GGCGACCTTGAGCAGCTAGT ATGCAGGTCGACGGATCTTT TCCGCTGCATAACCCTGCTT CGGGGTCATTATAGCGATTT

TTTCGGTATATCCATCCTTT TTCGCACGATATACAGGATT TTGCCAAAGGGTTCGTGTAG ACTTTCCTTGGTGTATCCAA CGGCGTCAGCCGGGCAGGAT

AGGTGAAGTAGGCCCACCCG CGAGCGGGTGTTCCTTCTTC ACTGTCCCTTATTCGCACCT GGCGGTGCTCAACGGGAATC CTGCTCTGCGAGGCTGGCCG

GCTACCGCCGGCGTAACAGA TGAGGGCAAGCGGATGGCTG ATGAAACCAAGCCAACCAGG AAGGGCAGCCCACCTATCAA GGTGTACTGCCTTCCAGACG

AACGAAGAGCGATTGAGGAA AAGGCGGCGGCGGCCGGCAT GAGCCTGTCGGCCTACCTGC TGGCCGTCGGCCAGGGCTAC AAAATCACGGGCGTCGTGGA

CTATGAGCACGTCCGCGAGC TGGCCCGCATCAATGGCGAC CTGGGCCGCCTGGGCGGCCT GCTGAAACTCTGGCTCACCG ACGACCCGCGCACGGCGCGG

TTCGGTGATGCCACGATCCT CGCCCTGCTGGCGAAGATCG AAGAGAAGCAGGACGAGCTT GGCAAGGTCATGATGGGCGT GGTCCGCCCGAGGGCAGAGC

CATGACTTTTTTAGCCGCTA AAACGGCCGGGGGGTGCGCG TGATTGCCAAGCACGTCCCC ATGCGCTCCATCAAGAAGAG CGACTTCGCGGAGCTGGTGA

AGTACATCACCGACGAGCAA GGCAAGACCGATCCCCGGGG ACCTGCATAGATCTGCCAGC CGCACTCGTCGCACGTCAGT GGTGCGGGCGGCTTGAGAAT

TCGTAATCATGTCATAGCTG TTTCCTGTGTGAAATTGTTA TCCGCTCACAATTCCACACA ACATACGAGCCGGAAGCATA AAGTGTAAAGCCTGGGGTGC

CTAATGAGTGAGCTAACTCA CATTAATTGCGTTGCGCTCA CTGCCCGCTTTCCAGTCGGG AAACCTGTCGTGCCAGCTGC ATTAATGAATCGGCCAACGC

GCGGGGAGAGGCGGTTTGCG TATTGGGCGCTCTTCCGCTT CCTCGCTCACTGACTCGCTG CGCTCGGTCGTTCGGCTGCG GCGAGCGGTATCAGCTCACT

CAAAGGCGGTAATACGGTTA TCCACAGAATCAGGGGATAA CGCAGGAAAGAACATGTGAG CAAAAGGCCAGCAAAAGGCC AGGAACCGTAAAAAGGCCGC

GTTGCTGGCGTTTTTCCATA GGCTCCGCCCCCCTGACGAG CATCACAAAAATCGACGCTC AAGTCAGAGGTGGCGAAACC CGACAGGACTATAAAGATAC

CAGGCGTTTCCCCCTGGAAG CTCCCTCGTGCGCTCTCCTG TTCCGACCCTGCCGCTTACC GGATACCTGTCCGCCTTTCT CCCTTCGGGAAGCGTGGCGC

TTTCTCATAGCTCACGCTGT AGGTATCTCAGTTCGGTGTA GGTCGTTCGCTCCAAGCTGG GCTGTGTGCACGAACCCCCC GTTCAGCCCGACCGCTGCGC

CTTATCCGGTAACTATCGTC TTGAGTCCAACCCGGTAAGA CACGACTTATCGCCACTGGC AGCAGCCACTGGTAACAGGA TTAGCAGAGCGAGGTATGTA

GGCGGTGCTACAGAGTTCTT GAAGTGGTGGCCTAACTACG GCTACACTAGAAGAACAGTA TTTGGTATCTGCGCTCTGCT GAAGCCAGTTACCTTCGGAA

AAAGAGTTGGTAGCTCTTGA TCCGGCAAACAAACCACCGC TGGTAGCGGTGGTTTTTTTG TTTGCAAGCAGCAGATTACG CGCAGAAAAAAAGGATCTCA

AGAAGATCCTTTGATCTTTT CTACGGGGTCTGACGCTCAG TGGAACGAAAACTCACGTTA AGGGATTTTGGTCATGAGAT TATCAAAAAGGATCTTCACC

TAGATCCTTTTGGTTCATGT GCAGCTCCATCAGCAAAAGG GGATGATAAGTTTATCACCA CCGACTATTTGCAACAGTGC CGTTGATCGTGCTATGATCG

ACTGATGTCATCAGCGGTGG AGTGCAATGTCGTGCAATAC GAATGGCGAAAAGCCGAGCT CATCGGTCAGCTTCTCAACC TTGGGGTTACCCCCGGCGGT

GTGCTGCTGGTCCACAGCTC CTTCCGTAGCGTCCGGCCCC TCGAAGATGGGCCACTTGGA CTGATCGAGGCCCTGCGTGC TGCGCTGGGTCCGGGAGGGA

CGCTCGTCATGCCCTCGTGG TCAGGTCTGGACGACGAGCC GTTCGATCCTGCCACGTCGC CCGTTACACCGGACCTTGGA GTTGTCTCTGACACATTCTG

GCGCCTGCCAAATGTAAAGC GCAGCGCCCATCCATTTGCC TTTGCGGCAGCGGGGCCACA GGCAGAGCAGATCATCTCTG ATCCATTGCCCCTGCCACCT

CACTCGCCTGCAGCCCGGTC GCCCGTGTCCATGAACTCGA TGGGCAGGTACTTCTCCTCG GCGTGGGACACGATGCCAAC ACGACGCTGCATCTTGCCGA

GTTGATGGCAAAGGTTCCCT ATGGGGTGCCGAGACACTGC ACCATTCTTCAGGATGGCAA GTTGGTACGCGTCGATTATC TCGAGAATGACCACTGCTGT

GAGCGCTTTGCCTTGGCGGA CAGGTGGCTCAAGGAGAAGA GCCTTCAGAAGGAAGGTCCA GTCGGTCATGCCTTTGCTCG GTTGATCCGCTCCCGCGACA

TTGTGGCGACAGCCCTGGGT CAACTGGGCCGAGATCCGTT GATCTTCCTGCATCCGCCAG AGGCGGGATGCGAAGAATGC GATGCCGCTCGCCAGTCGAT

TGGCTGAGCTCATGAGCGGA GAACGAGATGACGTTGGAGG GGCAAGGTCGCGCTGATTGC TGGGGCAACACGTGGAGCGG ATCGGGGATTGTCTTTCTTC

AGCTCGCTGATGATATGCTG ACGCTCAATGCCGTTTGGCC TCCGACTAACGAAAATCCCG CATTTGGACGGCTGATCCGA TTGGCACGGCGGACGGCGAA

TGGCGGAGCAGACGCTCGTC CGGGGGCAATGAGATATGAA AAAGCCTGAACTCACCGCGA CGTATCGATGTCGAGGTTCC TCAGGGGAGCCACCCCAGAG

AAGCCCTCGGAGCTGAGCGG AGCTATTTCCAAAGCCATGC CAGCTAGAGACAGTGCACAC CGCCAAGCATCTGCAAAACC CTCGCCTGGAGGGAAAGTGC

AATGTACGTACGCTGGTTTC CCTCCAGAAAGGGATTCTGC GGCTTCTCAACTTTGGAAGA AGAGGCGGAACGAACTGCTG TTTCAGCCTACTCATGTGAG

GAGGCTGGTCCTTTACCCTG ATGCCCGAGGGCATCAGGGT AAAGGACCAGCCTCGCCAAC TAGGAGCGCTACCAAGGCGA ACACAACCCAGGACTTGGTA

GAACCCTTCGCCGGAGAAGT TCAGACTCATCGGCATGAGC ACCCCTCGCCGCGCACTCGG 6260

**Nucleotide sequences of** **pKC1139-CR I S17:** The underlined sequences of pKC1139-CR II S16 are replaced by AGCCAGTCCATGCCGAAGCCTCGCGGGTCGACCTT
