# Supplementary material for: An Active Type I-E CRISPR-Cas System Identified in Streptomyces avermitilis
Source: PLoS One. 2016 Feb 22;11(2):e0149533. doi: 10.1371/journal.pone.0149533 (PMC4762764; doi:10.1371/journal.pone.0149533)
Supplement: S3 Table — (DOCX) [file pone.0149533.s006.docx]

**S3 Table. The conserved domains of the eight Cas proteins were analyzed by protein blast.**

| Locus on *S.avermitilis*  ATCC31267 | Conserved domain specific hits (accession number) | Superfamily | E-value |
| --- | --- | --- | --- |
| CAS_0001  (8983720-8983977) | Cas2_I-E(cd09755) | Cas2-I-E superfamily | 7.90e-24 |
| SAV_7537  (8984010-8985005) | Cas1_I-E(cd09719) | Cas_I-II-III superfamily | 6.65e-135 |
| SAV_7538  (8985002-8985670) | Cas6_I-E(cd09727) | Cas6_I-E superfamily | 4.43e-66 |
| SAV_7539  (8985667-8986503) | Cas5_I-E(cd09756) | Cas5_I superfamily | 5.99e-53 |
| SAV_7540  (8986500-8987678) | Cas7_I-E(cd09646) | Cas7_I-E superfamily | 6.82e-88 |
| SAV_7541  (8987744-8988430) | Cse2_I-E(cd09670) | Cse2_I-E superfamily | 3.94e-36 |
| SAV_7542  (8988473-8990044) | Cse1_I-E（cd09669） | Cse1 superfamily | 6.55e-126 |
| SAV_7543  (8990281-8993226) | Cas3_I-E（cd09641） | HDc and  ABC_ATPase superfamily | 3.39e-22 |
